# Development of a valid and reliable software customization model for SaaS quality through iterative method: perspectives from academia

**DOI:** 10.7717/peerj-cs.294

**Published:** 2020-09-21

**Authors:** Abdulrazzaq Qasem Ali, Abu Bakar Md Sultan, Abdul Azim Abd Ghani, Hazura Zulzalil

**Affiliations:** Department of Software Engineering and Information System, Faculty of Computer Science and Information Technology, Universiti Putra Malaysia, Serdang, Selangor, Malaysia

**Keywords:** Customization approaches, Content validity, Iterative method, Model development, Reliability study, SaaS quality, Software as a service

## Abstract

Despite the benefits of standardization, the customization of Software as a Service (SaaS) application is also essential because of the many unique requirements of customers. This study, therefore, focuses on the development of a valid and reliable software customization model for SaaS quality that consists of (1) generic software customization types and a list of common practices for each customization type in the SaaS multi-tenant context, and (2) key quality attributes of SaaS applications associated with customization. The study was divided into three phases: the conceptualization of the model, analysis of its validity using SaaS academic-derived expertise, and evaluation of its reliability by submitting it to an internal consistency reliability test conducted by software-engineer researchers. The model was initially devised based on six customization approaches, 46 customization practices, and 13 quality attributes in the SaaS multi-tenant context. Subsequently, its content was validated over two rounds of testing after which one approach and 14 practices were removed and 20 practices were reformulated. The internal consistency reliability study was thereafter conducted by 34 software engineer researchers. All constructs of the content-validated model were found to be reliable in this study. The final version of the model consists of 6 constructs and 44 items. These six constructs and their associated items are as follows: (1) Configuration (eight items), (2) Composition (four items), (3) Extension (six items), 4) Integration (eight items), (5) Modification (five items), and (6) SaaS quality (13 items). The results of the study may contribute to enhancing the capability of empirically analyzing the impact of software customization on SaaS quality by benefiting from all resultant constructs and items.

## Introduction

Software maintenance comprises a significant portion (70%) of the total software implementation costs (Lee, Park & Lim, 2013). According to Yang, Yoo & Jahng (2010), “more than 75% of the IT budget is spent just maintaining and running existing systems and software infrastructure”. The increase in development and operating costs, which was also one of the main reasons for the failure of application service provider (ASP) in the 1990s (De Miranda, 2010), is inevitable. As a result, the demand for a software as a service (SaaS) model is increasing because the costs of hardware, technology, maintenance, and tenant management are lower (Walraven, 2014; Walraven et al., 2014; Shen et al., 2011; Ali et al., 2017). Some problems, such as customization complexities (Walraven, 2014; Walraven et al., 2014; Guo et al., 2011; Al-Shardan & Ziani, 2015; Walraven, Truyen & Joosen, 2011), for the implementation of SaaS applications remain.

Customization is an essential requirement for providing the same application to different users (Walraven, 2014; Ali et al., 2018b), as they may have different business flow, interfaces, and data (Tsai, Zhong & Chen, 2016). Consequently for the hosts of SaaS applications, this requirement will pose quality challenges and risks (Al-Shardan & Ziani, 2015; Rolia et al., 2008). All SaaS application components are influenced by user-specific customization, including both functional and non-functional aspects of all layers of SaaS architecture (Tsai, Shao & Li, 2010).

Another complication is having to span multiple layers of SaaS architecture (Al-Shardan & Ziani, 2015). All SaaS application elements, including those with cross-layer relationships, must be customizable. Moreover, customization includes adjustments to the softwares source code that becomes highly complex in the SaaS model (Walraven, 2014; Walraven et al., 2014; Guo et al., 2011; Sun et al., 2008).

Changes in the requirements often occur after applications and services have been developed; therefore, runtime customization must be provided within the same software instance for different users (Walraven, 2014; Walraven et al., 2014; Ali et al., 2018a; Van Landuyt, Walraven & Joosen, 2015), and should not impact their privacy and the applications availability (Walraven, 2014; Van Landuyt, Walraven & Joosen, 2015). Generally, SaaS applications lack the customizability of on-premises applications (Yang, Yoo & Jahng, 2010), which would result in reduced software maintenance (Samir & Darwish, 2016; Xin & Levina, 2008). By contrast, frequent customization of the SaaS application would require a burdensome maintenance process and pose a significant challenge to scalability and cost-efficiency (Walraven et al., 2014; Van Landuyt, Walraven & Joosen, 2015). Therefore, application vendors should be cautious about their technical capacity when making customization assessments (Sun et al., 2008; Samir & Darwish, 2016), especially when customization impacts the crucial features of SaaS (Walraven, 2014; Walraven et al., 2014; Joha & Janssen, 2012; Espadas et al., 2013).

There is insufficient evidence in the available studies to assess the effect of software customization on SaaS attributes (Ali et al., 2019a). Accordingly, it is important that the type of customization be specified to assess the associated impact and risk (Chaumun et al., 2002) as the software quality are likely to be influenced by any change (Parthasarathy & Sharma, 2017; Parthasarathy & Sharma, 2016). Although several researchers have reported the need to consider the customization of SaaS applications, no clear effort has been made to categorize software customization types and practices in a multi-tenant context.

Accordingly, research is required to establish a clear model that considers: (1) generic software customization types and a list of common practices for each client in the SaaS multi-tenant context, and (2) key quality attributes associated with customization. Evidence of the content validity and reliability of the proposed model are reported in detail in this study. Two main calculations are considered for content validity: the item content validity index (I-CVI) of each customization practice and SaaS quality attributes, and the scale content validity index (S-CVI/Ave). Similarly, two quantities are evaluated to determine the internal consistency reliability of the model in this study: Cronbach’s alpha coefficient, and the corrected item-total correlation.

The structure of this manuscript is as follows. The next section discusses the related works. The third section presents the conceptualization of the model. The fourth section explains the methodology used, whereas the fifth section reports the results of the conducted study, followed by a discussion in the sixth section and threats to validity in the seventh section. Finally, conclusions and future work are presented in the eighth section.

## Related Work

This study presents an approach iteratively to develop, refine, and improve a software customization model for SaaS quality that was initially constructed in  (Ali et al., 2019b). The main components of this model are the customization types, common customization practices of each type, and quality attributes of SaaS applications associated with customization. To the best of our knowledge, no model based on these criteria has been developed and validated. However, in this section, we review the literature on generic SaaS customization options, followed by the literature on quality models for SaaS applications.

### SaaS customization

Different types of customization based on the layers of SaaS architecture and customization objects have been suggested (Li, Shi & Li, 2009; Tsai & Sun, 2013; Al-Shardan & Ziani, 2015). Li, Shi & Li (2009) illustrated five types of customization: GUI customization, service customization, process customization, data customization, and cross-layer customization. Tsai & Sun (2013) considered the customization of GUI, service, process, data, and QoS. Al-Shardan & Ziani (2015) defined three different types of SaaS customization: user interface, workflow, and access control.

Conversely, some studies classified SaaS customization based on how customization was performed. Tsai & Sun (2013) explained three types of customization: source code, composition, and configuration. Based on where the customizations are hosted and executed, the work of Müller et al. (2009) proposed three types of customization for multi-tenant SaaS applications: desktop integration, user-interface customization, and back-end customization.

Moreover, Kabbedijk & Jansen (2011) identified the types of customization in a tenant base. Customization was classified as segment variability and tenant-oriented variability: in the former, customization is performed based on the requirements of a tenant community, whereas in the latter, it is performed based on the specific requirements of a tenant. The most closely related studies are listed and summarized in Table 1.

**Table 1:** A summary of generic classification of SaaS Customization.

**References**	**Customization type**	**Based on**
Li, Shi & Li (2009)	GUI , service , process, data and cross-layer	SaaS architecture layers
Tsai & Sun (2013)	GUI, service, process, data and QoS	SaaS architecture layers
	Source code, composition and configuration	Manner of performing
Al-Shardan & Ziani (2015)	GUI, workflow and access control	SaaS architecture layers
Müller et al. (2009)	UI customization, desktop integration and back-end customization	Manner of performing
Kabbedijk & Jansen (2011)	Segment Variability and Tenant-oriented Variability	Tenant and Tenant’s community

As Table 1 indicates, although there were some generic customization approaches proposed for SaaS, they did not explicitly declare the common customization practices for each approach. In addition, several inconsistencies are found across all proposals. For example, the term “user interface customization” is used in both (Tsai & Sun, 2013; Müller et al., 2009), but with different meanings. Additionally, these proposals argued for the relevance of this approach, yet they did not consider reporting a comprehensive validation either from academia or industry.

### SaaS quality

Many studies have focused entirely on defining and identifying the quality attributes of SaaS applications. For instance, Khanjani et al. (2014) proposed a list of 33 quality attributes for SaaS and provided their definitions and Lee et al. (2009) proposed a comprehensive quality model for assessing SaaS cloud services. Based on ISO/IEC 9126 (Coallier, 2001), these authors identified characteristics and quality attributes and defined metrics to measure them. A systematic process was proposed by La & Kim (2009) to build a high-quality SaaS application, taking the main SaaS design criteria into consideration.

Duarte Filho et al. (2013) proposed a “SaaS Quality” method for evaluating the quality of SaaS applications. The SaaS quality model, based on ISO/IEC 9126 (Coallier, 2001) and IT management models (Publications Service Management, 2008; IT Governance Institute, 2007), was generated as a part of the proposed method. Another related study extracted the critical quality attributes for SaaS reusability and identified SaaS reusability metrics for every quality attribute (Samir & Darwish, 2016). Cancian et al. (2010) have customized software quality models to fit the SaaS context, classifying the SaaS quality criteria for products and processes and identifying quality attributes for each class.

Nadanam & Rajmohan (2012) proposed a QoS model for web services in cloud computing, similar to the work of (Lee et al., 2009). Some of these attributes have been included in the Lee model. However, these attributes only address a few relevant aspects; many other significant features remain to be considered. These two models focus largely on user perspectives. Table 2 summarizes all the SaaS quality models reported in this study. Although some works in Table 2 mention customizability as a quality attribute of SaaS applications, none of them focused on the quality attributes of SaaS applications associated with customization.

**Table 2:** A summary of proposed quality models for SaaS application.

**References**	**Quality attributes**	**Inspired by**
Khanjani et al. (2014)	Reliability, resiliency, Accuracy, Efficiency, Service response time, Stability, Functionality, Customizability, Suitability, Accessibility, Learnability, Commonality, Multi-tenancy, Operability,Serviceability, Robustness, Data Integrity, Data privacy, Adaptability, Extensibility, Flexibility, Scalability, Changeability, Composability, Maintainability, Security Management, Composability and Availability.	Service measurement index (CSMIC 2014)
Lee et al. (2009)	Efficiency, Reliability, scalability, availability and Reusability.	ISO/IEC 9126 (Coallier, 2001)
La & Kim (2009)	Supporting commonality, supporting multi tenant’s access, accessible via Internet, thin client model, Functionality, High Reusability, High Availability and High Scalability.	key characteristics desired properties of SaaS in (Espadas, Concha & Molina, 2008; Manford 2008)
Duarte Filho et al. (2013)	Functionality, Usability, Security, Performance, Support, Service Level, Portability	ISO/IEC 9126 (Coallier, 2001), ITIL v3 (Management 2008) and COBIT 4.1 (TGI 2007),
Samir & Darwish (2016)	Understandability, Modularity, Composability, Complexity, Customizability,reliability, Availability, Efficiency.	Literature analysis perfomed by the authors
Cancian et al. (2010)	Integrity,reliability, security, accessibility,requirements development and management, infrastructure capability, quality control, acquisition, testing, performance, utilization of standards, change control, interoperability,robustness, availability, maintenance, version control, technically competent in business, technically competent employees, prevision of continuity of service, scalability, help desk, process quality certification, governance,reputation.	Literature analysis perfomed by the authors
Nadanam & Rajmohan (2012)	Availability,reliability, scalability, efficiency,reusability, understandability, publicity, adaptability and composability.	Literature analysis perfomed by the authors

## Conceptual Model

Based on a systematic mapping study (SMS) conducted by Ali et al. (2019), the proposed model was initially constructed from 46 customization practices and 13 quality attributes in the SaaS multi-tenant context. Each of the investigated customization practices was assigned to a customization approach (personalization, configuration, composition, modification, integration, and extension). These approaches were inferred from the most popular software customization proposals (Ali et al., 2019).

**Figure 1:**
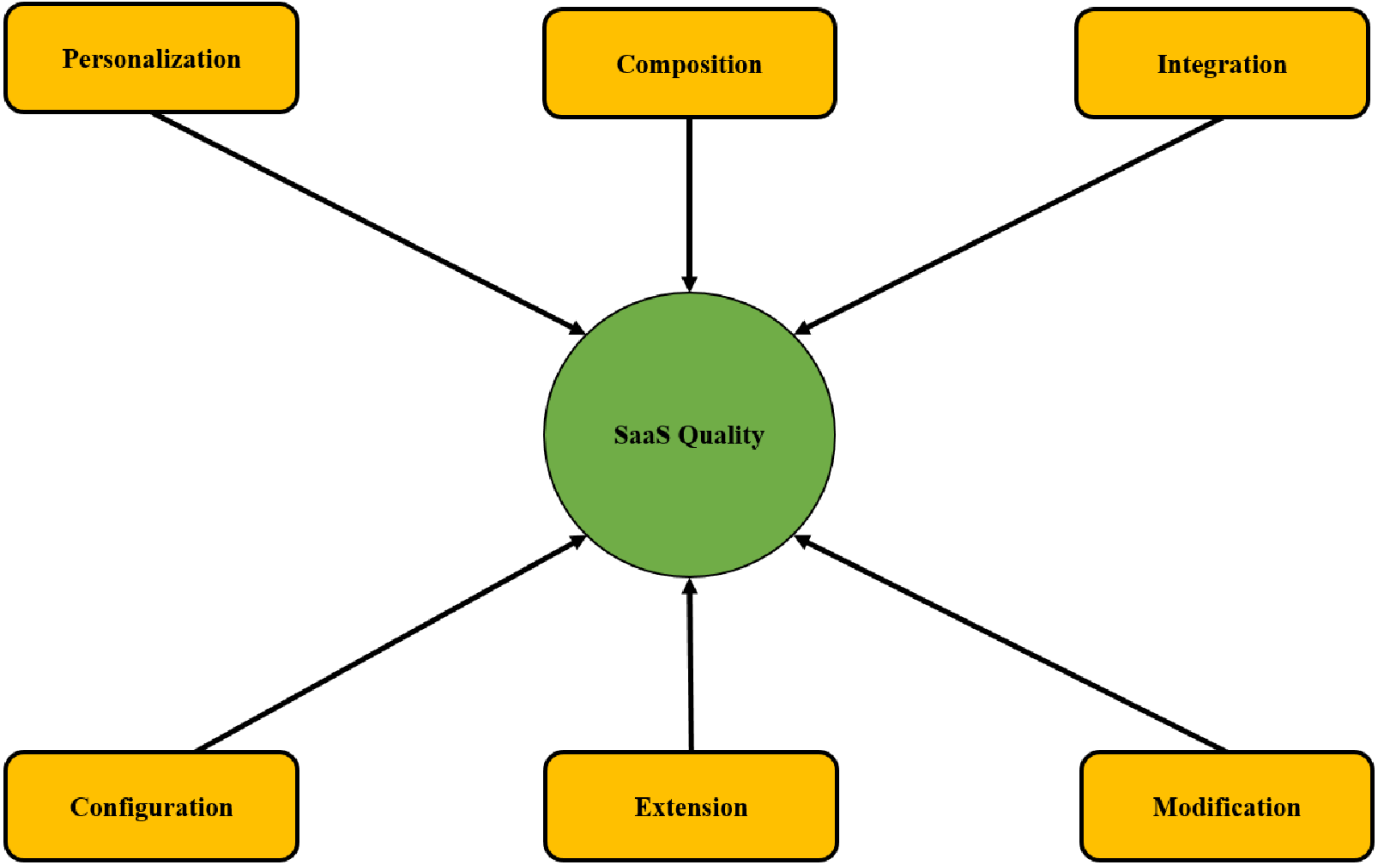
Proposed model of this study.

The model presented in this study, as shown in Fig. 1, demonstrates the concept of all the approaches and SaaS quality. The purpose of the conceptual model is to analyze the variables in this study comprehensively.

### Personalization approach

The personalization approach refers to all solutions that provide transparent customization without needing to inform users (Gilmore & Pine, 1997; Mathiassen & Sandberg, 2014; Sunikka & Bragge, 2008). Personalization involves gathering and analyzing datasets correlated to individuals and/or groups (Tsai, Shao & Li, 2010; Fan et al., 2015; Tsai, Huang & Shao, 2011; Truyen et al., 2012) accurately to implement services based on their current and common preferences (Tsai, Shao & Li, 2010; Fan et al., 2015; Truyen et al., 2012). Moreover, a set of potential services is offered by publicly available pre-structured templates from SaaS providers (Fan et al., 2015). The main data sources for personalization may be tenant or tenant communities (Tsai, Shao & Li, 2010).

Recommendation mechanisms are often used with this approach to propose suitable services according to preferences, profiles, data, and service directories of the users (Tsai, Shao & Li, 2010; Fan et al., 2015). The personalization approach also considers the meaning (semantics) of user and community data (Tsai, Shao & Li, 2010; Fan et al., 2015) by employing runtime behavior adaptation facilities to adapt the behavior of SaaS applications to the performance context (Truyen et al., 2012; Xiaojun, Yongqing & Lanju, 2013; Aulbach et al., 2011). A summary of common customization practices related to personalization in the context of multi-tenant SaaS applications is given in Table 3.

**Table 3:** Multi-tenant SaaS customization practices of Personalization approach.

**Id**	**Customization practice**	**References**
Par 1	Tenants profile	Tsai, Shao & Li (2010), Fan et al. (2015), Tsai, Huang & Shao (2011), Truyen et al. (2012)
Par 2	Tenants preferences	Tsai, Shao & Li (2010), Fan et al. (2015), Truyen et al. (2012)
Par 3	Tenants behavioral activities	Fan et al. (2015), Truyen et al. (2012)
Par 4	Service directory	Fan et al. (2015)
Par 5	Recommendation mechanism	Tsai, Shao & Li (2010), Fan et al. (2015)
Par 6	Semantics data	Tsai, Shao & Li (2010), Fan et al. (2015)
Par 7	Runtime personalization	Truyen et al. (2012), Xiaojun, Yongqing & Lanju (2013), Aulbach et al. (2011)
Par 8	Tenants and Tenants communities (Info source)	Tsai, Shao & Li (2010)

### Configuration approach

According to the configuration approach, solutions offer a predefined setting for the alteration of application functions within a predefined scope (Sun et al., 2008; Brehm, Heinzl & Markus, 2001; Parhizkar, 2016; Davenport, 1998). Diversity is usually maintained by establishing predefined parameters, options, and components, treating each user individually (Xin & Levina, 2008; Salih & Zang, 2016; Kabbedijk & Jansen, 2011). Each SaaS tenant has to be able to configure the application by employing techniques to modify the functions of the applications within established limits (Xin & Levina, 2008; Zhang et al., 2007; Li, Shi & Li, 2009). Meanwhile, SaaS providers have to develop sets of services and plugins with which tenants perform configurations (Zhao et al., 2014; Mohamed et al., 2014). This type of SaaS application must enable tenants to create or select features based on the template repository (Tsai, Shao & Li, 2010; Tsai, Huang & Shao, 2011; Saleh, Fouad & Abu-Elkheir, 2014; Ralph, 2008; Chen, Li & Kong, 2013; Ruehl & Andelfinger, 2011; Tsai & Sun, 2013).

A set of components, which accommodate a variety of tenant needs, is provided in the application template. By selecting a relevant component set, tenants can personalize each customization point (Shahin, 2014; Mietzner & Leymann, 2008). When a tenant wishes to subscribe to the SaaS application, the capabilities of each feature within the system are analyzed to determine whether they ought to be incorporated into the application (Mohamed et al., 2014; Ying et al., 2010). All configurations established by the tenants must be adapted during the applications runtime (Xin & Levina, 2008; Gey, Landuyt & Joosen, 2015; Moens & De Turck, 2014; Shi et al., 2009). In addition, a disabling or excluding option for some features could be provided (Nguyen, Colman & Han, 2016; Moens et al., 2012). Table 4 summarizes the common customization practices of the configuration approach in the context of multi-tenant SaaS applications.

**Table 4:** Multi-tenant SaaS customization practices of Configuration approach.

**Id**	**Customization practice**	**References**
Con 1	Pre-defined parameters and options	Xin & Levina (2008), Salih & Zang (2016), Kabbedijk & Jansen (2011)
Con 2	Tenant configuration Interface	Xin & Levina (2008), Zhang et al. (2007), Li, Shi & Li (2009)
Con 3	Provider plugins	Zhao et al. (2014), Mohamed et al. (2014)
Con 4	Customization templates	Tsai, Shao & Li (2010), Tsai, Huang & Shao (2011), Saleh, Fouad & Abu-Elkheir (2014), Ralph (2008), Chen, Li & Kong (2013), Ruehl & Andelfinger (2011), Tsai & Sun (2013)
Con 5	Template repository	Tsai, Huang & Shao (2011), Saleh, Fouad & Abu-Elkheir (2014), Tsai & Sun (2013)
Con 6	Customization point	Shahin (2014), Mietzner & Leymann (2008)
Con 7	Feature selection	Mohamed et al. (2014), Ying et al. (2010)
Con 8	Runtime Configuration	Xin & Levina (2008), Gey, Landuyt & Joosen (2015), Moens & De Turck (2014), Shi et al. (2009)
Con 9	Features deactivation	Nguyen, Colman & Han (2016), Moens et al. (2012)

### Composition approach

In this approach, the multiple interacting components of the SaaS application are consolidated and new components can be shared between tenants and end-users (Saleh, Fouad & Abu-Elkheir, 2014; Moens et al., 2012; Moens, Dhoedt & De Turck, 2015; Liu et al., 2010; Rico et al., 2016; Ruehl, Wache & Verclas, 2013; Makki et al., 2016). Different components of the SaaS applications that collaborate must be composed during runtime (Moens et al., 2012; Moens, Dhoedt & De Turck, 2015; Kumara et al., 2013; Mietzner, Leymann & Papazoglou, 2008; Lee & Choi, 2012). The final composition must take into consideration the subcomponents of the core application (Kumara et al., 2013; Schroeter et al., 2012; Kumara et al., 2015). The composition approach enables simplification of the consolidated SaaS components (Shahin, 2014; Moens et al., 2012; Gey et al., 2014) as the relationships and dependencies between them are considered (Li, Shi & Li, 2009; Shahin, 2014; Moens, Dhoedt & De Turck, 2015). Table 5 summarizes the common customization practices of the composition approach in the context of multi-tenant SaaS applications.

**Table 5:** Multi-tenant SaaS customization practices of Composition approach.

**Id**	**Customization practice**	**References**
Com 1	Components consolidation and sharing	Saleh, Fouad & Abu-Elkheir (2014), Moens et al. (2012), Moens, Dhoedt & De Turck (2015), Liu et al. (2010), Rico et al. (2016), Ruehl, Wache & Verclas (2013), Makki et al. (2016)
Com 2	Runtime composition	Moens et al. (2012), Moens, Dhoedt & De Turck (2015), Kumara et al. (2013), Mietzner, Leymann & Papazoglou (2008), Lee & Choi (2012)
Com 3	Subcomponents composition	Kumara et al. (2013), Schroeter et al. (2012), Kumara et al. (2015)
Com 4	Decomposition	Shahin (2014), Moens et al. (2012), Gey et al. (2014)
Com 5	Components relationships	Li, Shi & Li (2009), Shahin (2014), Moens, Dhoedt & De Turck (2015)

### Extension approach

SaaS application can be extended by adding custom code to be compiled during runtime (Saleh, Fouad & Abu-Elkheir, 2014; Mietzner & Leymann, 2008; Correia, Penha & Da Cruz, 2013). Furthermore, a set of extension points, which permit a customized service to be plugged in, must be provided (Mietzner & Leymann, 2008; Correia, Penha & Da Cruz, 2013; Moens & De Turck, 2014; Salih & Zang, 2012). The SaaS provider should also supply an open platform and an API, which allows developers to compile custom code (replacements for existing objects or extensions to them) into the business object layers of the application (Zhao et al., 2014; Müller et al., 2009). Any extension to a SaaS application may be public or accessible only by an individual tenant (Aulbach et al., 2011). Table 6 summarizes the common customization practices of the extension approach in the context of multi-tenant SaaS.

**Table 6:** Multi-tenant SaaS customization practices of Extension approach.

**Id**	**Customization practice**	**References**
Ext 1	Custom code insertion	Saleh, Fouad & Abu-Elkheir (2014), Mietzner & Leymann (2008), Correia, Penha & Da Cruz (2013)
Ext 2	Extension points	Mietzner & Leymann (2008), Correia, Penha & Da Cruz (2013)
Ext 3	Runtime extension	Moens & De Turck (2014), Salih & Zang (2012)
Ext 4	Extension interface	Zhao et al. (2014), Müller et al. (2009)
Ext 5	Replacements of existing code	Müller et al. (2009)
Ext 6	Private/public extension	Aulbach et al. (2011)

### Integration approach

In this case, the SaaS application must be capable of incorporating extra services via external SaaS providers (Aulkemeier et al., 2016; Sun et al., 2007; Almorsy, Grundy & Ibrahim, 2012; Scheibler, Mietzner & Leymann, 2008). Most SaaS service customers assume that the SaaS application will merge easily with their in-house systems (Müller et al., 2009; Aulkemeier et al., 2016; Sun et al., 2007; Scheibler, Mietzner & Leymann, 2008; Zhang, Liu & Meng, 2009). However, this integration must consider nonfunctional elements, such as security controls, which should be accommodated by the applications architecture (Sun et al., 2007; Almorsy, Grundy & Ibrahim, 2012), at both design time and runtime (Aulkemeier et al., 2016; Sun et al., 2007).

Integration platforms may present a service framework, responsible for assimilating services, and process frameworks, through which business processes can be executed (Aulkemeier et al., 2016; Sun et al., 2007). Any additional services from third-party SaaS providers must employ different programming languages and run in different environments (Scheibler, Mietzner & Leymann, 2008). In some cases, code or scripts will be utilized to incorporate these services (Aulkemeier et al., 2016). Incorporation requires an integration interface (Aulkemeier et al., 2016; Sun et al., 2007), synchronization toolkits, and data retrieval mechanisms to respond to the demands posed by integration (Sun et al., 2007; Zhang, Liu & Meng, 2009). In this study, the common customization practices related to the integration approach in the context of multi-tenant SaaS applications are summarized in Table 7.

**Table 7:** Multi-tenant SaaS customization practices of Integration approach.

**Id**	**Customization practice**	**References**
Int 1	Integration with another SaaS	Mohamed et al. (2014), Aulkemeier et al. (2016), Sun et al. (2007), Almorsy, Grundy & Ibrahim (2012), Scheibler, Mietzner & Leymann (2008)
Int 2	Integration with other on-premise applications	Müller et al. (2009), Aulkemeier et al. (2016), Sun et al. (2007), Scheibler, Mietzner & Leymann (2008), Zhang, Liu & Meng (2009)
Int 3	Non-functional integration	Sun et al. (2007), Almorsy, Grundy & Ibrahim (2012)
Int 4	Runtime integration	Aulkemeier et al. (2016), Sun et al. (2007)
Int 5	Service & process integration	Aulkemeier et al. (2016), Sun et al. (2007)
Int 6	Integration of different PL applications	Scheibler, Mietzner & Leymann (2008)
Int 7	Third party code injection	Aulkemeier et al. (2016)
Int 8	Integration interface	Aulkemeier et al. (2016), Sun et al. (2007)
Int 9	Synchronization & data retrieval tools	Sun et al. (2007), Zhang, Liu & Meng (2009)

### Modification approach

This approach refers to techniques and solutions that alter the design or other functional requirements of the application by changing part of its source code (Luo & Strong, 2004; Haines, 2009). The modifications must consider the allocation of resources to take the customized code into account, thereby ensuring operational cost-efficiency regarding maintenance and resource sharing among tenants (Sun et al., 2008; Moens & De Turck, 2014; Helmich et al., 2009). SaaS vendors must manage all elements of customization codes for any individual tenant without developing unique versions for each (Sun et al., 2008). As a result, they should alter the code of the application when identical customizations are made by a considerable number of tenants (Sun et al., 2008; Moens & De Turck, 2014).

Runtime code changes must consider the interrelationship between different functions, as one function can depend on one or several other functions simultaneously (Dong et al., 2010). Namespaces, inheritance, and polymorphism are often used to implement source code customizations in this case (Lee & Choi, 2012). Source-code modifications are made by adding or deleting methods or attributes, or by changing the current implementation methods of the object (Ziani & AlShehri, 2015; Kong, Li & Zheng, 2010). Table 8 summarizes the common customization practices of the modification approach in the context of multi-tenant SaaS applications.

**Table 8:** Multi-tenant SaaS customization practices of Modification approach.

**Id**	**Customization practice**	**References**
Mod 1	Source code modifications	Sun et al. (2008), Moens & De Turck (2014), Helmich et al. (2009)
Mod 2	Resources allocation for customized code	Sun et al. (2008), Moens & De Turck (2014)
Mod 3	Individual tenant basis	Sun et al. (2008)
Mod 4	Identical customizations	Sun et al. (2008), Moens & De Turck (2014)
Mod 5	Runtime Modification	Moens & De Turck (2014)
Mod 6	Dependency relationship of modified functions	Dong et al. (2010)
Mod 7	Namespaces, inheritance, and polymorphism	Lee & Choi (2012)
Mod 8	Add or changes methods or attributes	Ziani & AlShehri (2015), Kong, Li & Zheng (2010)
Mod 9	Deletion of custom objects, methods, or attributes	Ziani & AlShehri (2015), Kong, Li & Zheng (2010)

### SaaS quality

The list of SaaS quality attributes in the proposed customization solutions for SaaS applications was provided in (Ali et al., 2019), but the attributes were not defined. Therefore, in this work, we focus on the definitions provided by (Khanjani et al., 2014), which have been evaluated and validated in a Ph D dissertation (Khanjani, 2015). Moreover, these definitions were compared with those available in the literature provided by other SaaS quality models.

The identification and definitions of the quality attributes that play an important role in SaaS customization or could be influenced by customization are presented in Table 9.

**Table 9:** Quality attributes of SaaS applications associated with customization.

**Id**	**Quality attribute**	**Defination**	**References**
QA 1	**Multi-tenancy**	SaaS services can support instances of simultaneous access by multiple users for multiple tenants.	Khanjani et al. (2014), La & Kim (2009)
QA 2	**Scalability**	SaaS providers can manage growth or decline in the level of services.	Khanjani et al. (2014), Lee et al. (2009), Nadanam & Rajmohan (2012), Zia & Khan (2012), Akojwar et al. (2012), CSMIC (2014)
QA 3	**Availability**	SaaS services can function within a specific time to satisfy users needs.	Khanjani et al. (2014), Lee et al. (2009), Nadanam & Rajmohan (2012), Akojwar et al. (2012), CSMIC (2014), Cancian et al. (2010), Alhamad, Dillon & Chang (2010)
QA 4	**Reliability**	SaaS application maintains operating and functioning under given conditions without failure within a given time period.	Khanjani et al. (2014), Lee et al. (2009), Nadanam & Rajmohan (2012), Akojwar et al. (2012), CSMIC (2014), Cancian et al. (2010), Alhamad, Dillon & Chang (2010)
QA 5	**Maintainability**	Modifications to the application are made by SaaS provider to retain it in the condition of good repair.	Khanjani et al. (2014), CSMIC (2014), Cancian et al. (2010)
QA 6	**Security**	The effectiveness of SaaS provider’s controls on service data, access to the services, and the physical facilities from which service are provided.	Khanjani et al. (2014), CSMIC (2014)
QA 7	**Usability**	The ease with which SaaS service can be used to achieve tenant-specific-goal.	Khanjani et al. (2014), Alhamad, Dillon & Chang (2010)
QA 8	**Interoperability**	SaaS service can easily interact with other services from the same SaaS provider or other providers.	Khanjani et al. (2014), CSMIC (2014), Cancian et al. (2010)
QA 9	**Efficiency**	SaaS services effectively utilize resources to perform their functions.	Khanjani et al. (2014), Lee et al. (2009), Nadanam & Rajmohan (2012), Akojwar et al. (2012)
QA 10	**Functionality**	SaaS application provides an extensive set of features.	Khanjani et al. (2014), CSMIC (2014)
QA 11	**Accessibility**	SaaS services are operable by users with different disabilities.	Khanjani et al. (2014), CSMIC (2014), Cancian et al. (2010)
QA 12	**Commonality**	SaaS services possess common features and are amenable to reuse by multiple users.	Khanjani et al. (2014), La & Kim (2009), Lee et al. (2009), Nadanam & Rajmohan (2012)
QA 13	**Response time**	SaaS application adheres to a defined time limit between service request and service response.	Khanjani et al. (2014), CSMIC (2014), Salama et al. (2012), Badidi (2013), Song et al. (2012), He et al. (2012), Wang et al. (2011)

**Figure 2:**
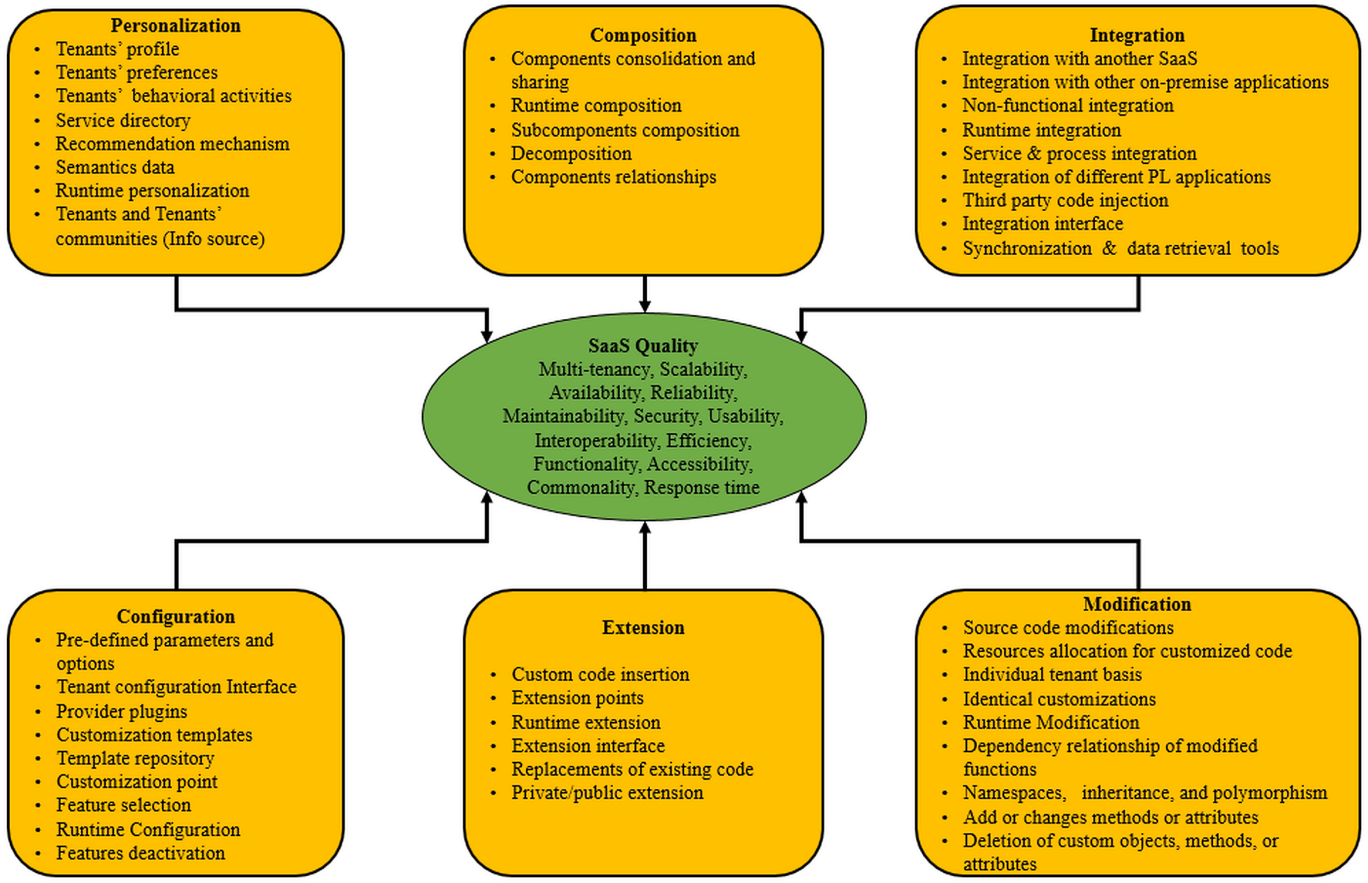
Conceptual model of this study.

All the customization practices for each approach and the quality attributes associated with the relevant SaaS applications are presented in Fig. 2. In this study, all customization approaches are variables that may affect the quality of SaaS applications. In the remaining sections of this study, customization practices and quality attributes are labeled as items, while approaches and SaaS quality are labeled as “constructs”.

## Methodology

**Figure 3:**
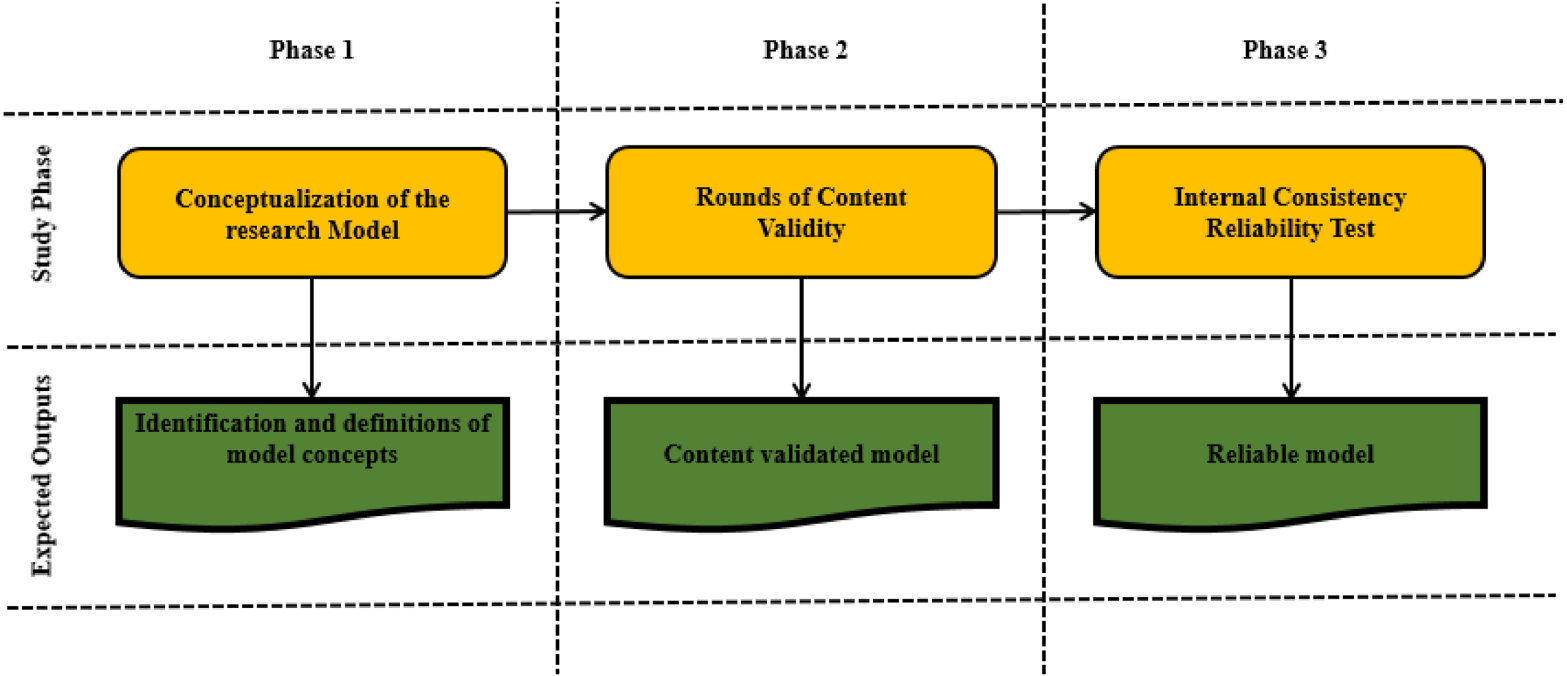
Methodology phases of this study.

The methodology of this study is composed of three main phases, as indicated in Fig. 3. The first phase is the development of the customization model concept for SaaS quality, as presented in the previous section. The second and third phases consider the content validity and reliability of the model.

### Rounds of content validity

Content validity is a vital topic for high-quality measurement (Wynd, Schmidt & Schaefer, 2003). It holds that each item has a requisite sample of aspects that depicts the construct of interest (Cohen, Manion & Morrison, 2002). In this study, this quantity was evaluated to validate the conceptual model.

Content validity is generally determined based on the opinion of experts, who analyze if the model or instrument correctly depicts its concept (Bell, Bryman, & Harley, 2015; Hair et al., 2015), in the field. To validate the conceptual model, a questionnaire was elaborated and provided to researchers who had previous experience in the SaaS customization field. These researchers were identified through an extensive systematic mapping study and selected based on their published papers and affinity with this study, 224 researchers were identified (Ali et al., 2019). A cover letter describing the objective of the questionnaire and asking some personal information on the background of experts was attached.

As the available literature on the classification and identification of software customization approaches and related quality attributes in the SaaS multi-tenant context is insufficient, we conducted iterative content validity evaluation as recommended by (Polit & Beck, 2006; Parratt et al., 2016; Harris & Holloway, 2012). While designing the data analysis for each round, we primarily followed the content validity index (CVI) method, which is the common method for this purpose (Bhatti & Ahsan, 2017), as guided by (Polit & Beck, 2006). The popularity of CVI is not only limited to educational, psychological, or nursing research, but also to other disciplines or research areas, such as researches in software engineering and information systems (Bhatti & Ahsan, 2017; Yilmaz et al., 2017; Wang et al., 2019). In this study, two quantities were calculated (Polit & Beck, 2006):

1. The item content validity index (I-CVI) for each item.
2. The scale content validity index (S-CVI/Ave), which is an average of the I-CVIs for each construct.

Lynn (1986) suggests that at least three experts should be present to evaluate the model; however, more than ten experts would probably be unnecessary (Polit & Beck, 2006). Other scholars mention that at least five experts should be sufficient to validate the model (Zamanzadeh et al., 2015). Questionnaires that queried the relevance of each item with respect to its construct were, therefore, sent to a group of experts. As recommended by (Polit & Beck, 2006; Lynn, 1986; Davis, 1992), the respondent replied according to a 4-point ordinal scale in which 1, 2, 3 and 4 respectively corresponded to “not relevant”, “somewhat relevant”, “quite relevant”, and “very relevant”. The experts were also requested to provide further comments about each item and construct and about the overall model, including recommendations for improvement.

After each round (after at least five experts had replied to the questionnaire), the inputs and suggestions were analyzed. Any item that was deemed unclear or did not meet the I-CVI criteria was either revised or removed. The rounds were suspended when the S-CVI and I-CVI criteria were achieved:

1. I-CVI of 1.00 with 3 to 5 experts (Polit & Beck, 2006; Lynn, 1986).
2. I-CVI of 0.78 or higher for 6 to 10 experts (Polit & Beck, 2006; Lynn, 1986).
3. S-CVI of 0.80 or higher (Polit & Beck, 2006; Yamada et al., 2010).

Our intention in round 1 was to revise the items that did not meet the I-CVI criteria rather than deleting them. The deletion of invalid items or constructs was left to the subsequent rounds. This strategy, therefore, allowed most of the items to be assessed more than one time.

Furthermore, the CVI analysis in all rounds had to be supplemented by computation of the Kappa coefficient to remove possible chance agreement among the experts (Shrotryia & Dhanda, 2019; Polit & Beck, 2006). The evaluation criteria for Kappa are fair = k of 0.40–0.59, good = k of 0.60–0.74, and excellent = k > 0.74 (Zamanzadeh et al., 2014; Shrotryia & Dhanda, 2019; Polit & Beck, 2006).

### Reliability study

After the content validity was established, a study was conducted to determine the reliability of the model. Thirty-four respondents from software engineering research groups, familiar with SaaS applications, were purposively sampled. They were from four Malaysian universities, namely Universiti Putra Malaysia, Universiti Kebangsaan Malaysia, Universiti Malaysia Pahang, and Universiti Tenaga Nasional.

The reliability of the measured items used in the survey was examined using Cronbachs alpha internal consistency test. Its results range from 0 to 1, in which high numbers indicate high reliability. Values greater than 0.9 are excellent; between 0.8 and 0.9 are good; between 0.7 and 0.8 are acceptable; between 0.6 and 0.7 are questionable, and below 0.6 are poor (Sekaran & Bougie, 2016). The reliability of the research instrument or model is related to its consistency and stability (Sekaran & Bougie, 2016; Alkawsi, ALI & Alghushami, 2018). The reliability of the model was assessed using three quantities:

1. Cronbach’s alpha value for each construct must be 0.70 or above.
2. Corrected item-total correlation should exceed 0.2.
3. Cronbachs Alpha if Item deleted must be lower than that of Cronbach’s alpha for a construct.

## Results

The results of the content validity evaluation and consistency tests are reported in this section.

### Rounds of content validity

We conducted two evaluation rounds for content validity between February 2019 and June 2019, starting with version 1 of the model produced in the conceptualization phase. It was revised after each round, generating versions 2 and 3. The versions 1, 2, and 3 questionnaires are provided in Appendices A–C.

In round 1, the questionnaire was sent to the first 100 researchers identified by Ali et al. (2019); only five experts replied and completed the content validity questionnaire. Therefore, in round 2, we considered sending it to all the researchers identified by Ali et al. (2019) (including all the researchers who were addressed in round 1); only six experts replied. Tables 10 and 11 contain the basic research-related information of the experts who participated in rounds 1 and 2. Due to the satisfying level of consensus indicated by the I-CVI and S-CVI scores after the analysis of round 2, it was determined that an additional round was unnecessary; therefore, data collection was suspended.

**Table 10:** Basic research-related information of the experts participated in round 1.

**No**	**Designation**	**Research expertise**	**Experience**
1	Researcher	Software Engineering, Software Systems	>5
2	Associate Professor	Software Engineering	<5
3	Professor	Software Engineering, Software Tools, Model-driven Development	>5
4	Associate Professor	Software engineering	>5
5	Researcher	Software engineering, big data, AI	<5

**Table 11:** Basic research-related information of the experts participated in round 2.

**No**	**Designation**	**Research expertise**	**Experience**
1	Assistant Professor	Software Engineering	>5
2	Professor	Software Engineering	>5
3	Researcher	Software Engineering, Distributed & Cloud Computing	>5
4	Researcher	Software Engineering, Machine Learning	<5
5	Associate Professor	Software Engineering, Cloud Computing	>5
6	Associate Professor	Software engineering	>5

Table 12 demonstrates the level of consensus for each of the items in the two rounds as well as the initial 59 items and 7 constructs of round 1, and 56 items and 7 constructs of round 2. These items were deleted in round 1 for the following reasons:

**Table 12:** Results of I-CVI, S-CVI and Kappa within content validity rounds.

**Construct**	**Round_1**				**Round_2**			
	**Item_1**	**I-CVI_1**	**Kappa**	**S-CVI_1**	**Item_2**	**I-CVI_2**	**Kappa**	**S-CVI_2**
Personalization ^a^	Per 1	0.4	0.13	0.8	Per 1	0.67	0.56	0.72
	Per 2	0.8	0.76		Per 2	1	1.00	
	Per 3	0.8	0.76		Per 3	0.5	0.27	
	Per 4	0.8	0.76		Per 4	0.67	0.56	
	Per 5	0.8	0.76		Per 5	0.67	0.56	
	Per 6	0.8	0.76		Per 6	0.67	0.56	
	Per 7	1	1.00		Per 7	0.83	0.82	
	Per 8	1	1.00		Per 8	0.83	0.82	
Configuration	Con 1	0.4	0.13	0.8	Con 1	1	1.00	0.958
	Con 2	0.8	0.76		Con 2	1	1.00	
	Con 3	1	1.00		Con 3	1	1.00	
	Con 4	1	1.00		Con 4	1	1.00	
	Con 5	1	1.00		Con 5	0.83	0.82	
	Con 6	0.8	0.76		Con 6	0.83	0.82	
	Con 7	0.8	0.76		Con 7	1	1.00	
	Con 8	0.8	0.76		Con 8	1	1.00	
	Con 9	0.6	0.42					
Composition	Com 1	1	1.00	0.84	Com 1	1	1.00	0.86^b^
	Com 2	1	1.00		Com 2	0.83	0.82	
	Com 3	0.8	0.76		Com 3	1	1.00	
	Com 4	0.4	0.13		Com 4	0.5	0.27	
	Com 5	1	1.00		Com 5	1	1.00	
Extension	Ext 1	1	1.00	0.9	Ext 1	1	1.00	0.91
	Ext 2	1	1.00		Ext 2	0.83	0.82	
	Ext 3	0.6	0.42		Ext 3	0.83	0.82	
	Ext 4	1	1.00		Ext 4	1	1.00	
	Ext 5	0.8	0.76		Ext 5	1	1.00	
	Ext 6	1	1.00		Ext 6	0.83	0.82	
Integration	Int 1	1	1.00	0.75	Int 1	1	1.00	0.92
	Int 2	1	1.00		Int 2	0.83	0.82	
	Int 3	1	1.00		Int 3	0.83	0.82	
	Int 4	0.6	0.42		Int 4	1	1.00	
	Int 5	0.8	0.76		Int 5	1	1.00	
	Int 6	0.4	0.13		Int 6	1	1.00	
	Int 7	0.6	0.42		Int 7	0.83	0.82	
	Int 8	0.6	0.42		Int 8	1	1.00	
	Int 9	0.8	0.76		Int 9	0.83	0.82	
Modification	Mod 1	0.8	0.76	0.77	Mod 1	0.83	0.82	0.809^c^
	Mod 2	0.6	0.42		Mod 2	1	1.00	
	Mod 3	0.8	0.76		Mod 3	0.83	0.82	
	Mod 4	0.8	0.76		Mod 4	0.83	0.82	
	Mod 5	0.8	0.76		Mod 5	0.67	0.56	
	Mod 6	0.8	0.76		Mod 6	0.67	0.56	
	Mod 7	0.6	0.42		Mod 7	0.83	0.82	
	Mod 8	1	1.00					
	Mod 9	0.8	0.76					
SaaS Quality	QA 1	1	1.00	0.76	QA 1	1	1.00	0.98
	QA 2	1	1.00		QA 2	1	1.00	
	QA 3	1	1.00		QA 3	1	1.00	
	QA 4	0.8	0.76		QA 4	1	1.00	
	QA 5	0.6	0.42		QA 5	1	1.00	
	QA 6	0.6	0.42		QA 6	1	1.00	
	QA 7	0.6	0.42		QA 7	1	1.00	
	QA 8	0.8	0.76		QA 8	1	1.00	
	QA 9	0.8	0.76		QA 9	1	1.00	
	QA 10	0.8	0.76		QA 10	1	1.00	
	QA 11	0.6	0.42		QA 11	0.83	0.82	
	QA 12	0.8	0.76		QA 12	1	1.00	
	QA 13	0.6	0.42		QA 13	1	1.00	

**Notes.**

a Items and costructs with red color were removed from the Model.

b S-CVI of Composition construct after remvoing invalid items is 0.95.

c S-CVI of Modification construct after remvoing invalid items is 0.86.

1. Item Con 1 was removed as it was adequately measured by item Con 6, thus item Con 6 was retained as its I-CVI (0.8) was higher than item Con 1 (0.4).
2. Item Mod 7 was removed as it was applicable to all software developed with object-oriented approach.
3. Item Mod 9 was merged with item Mod 8 as they complement each other.

**Figure 4:**
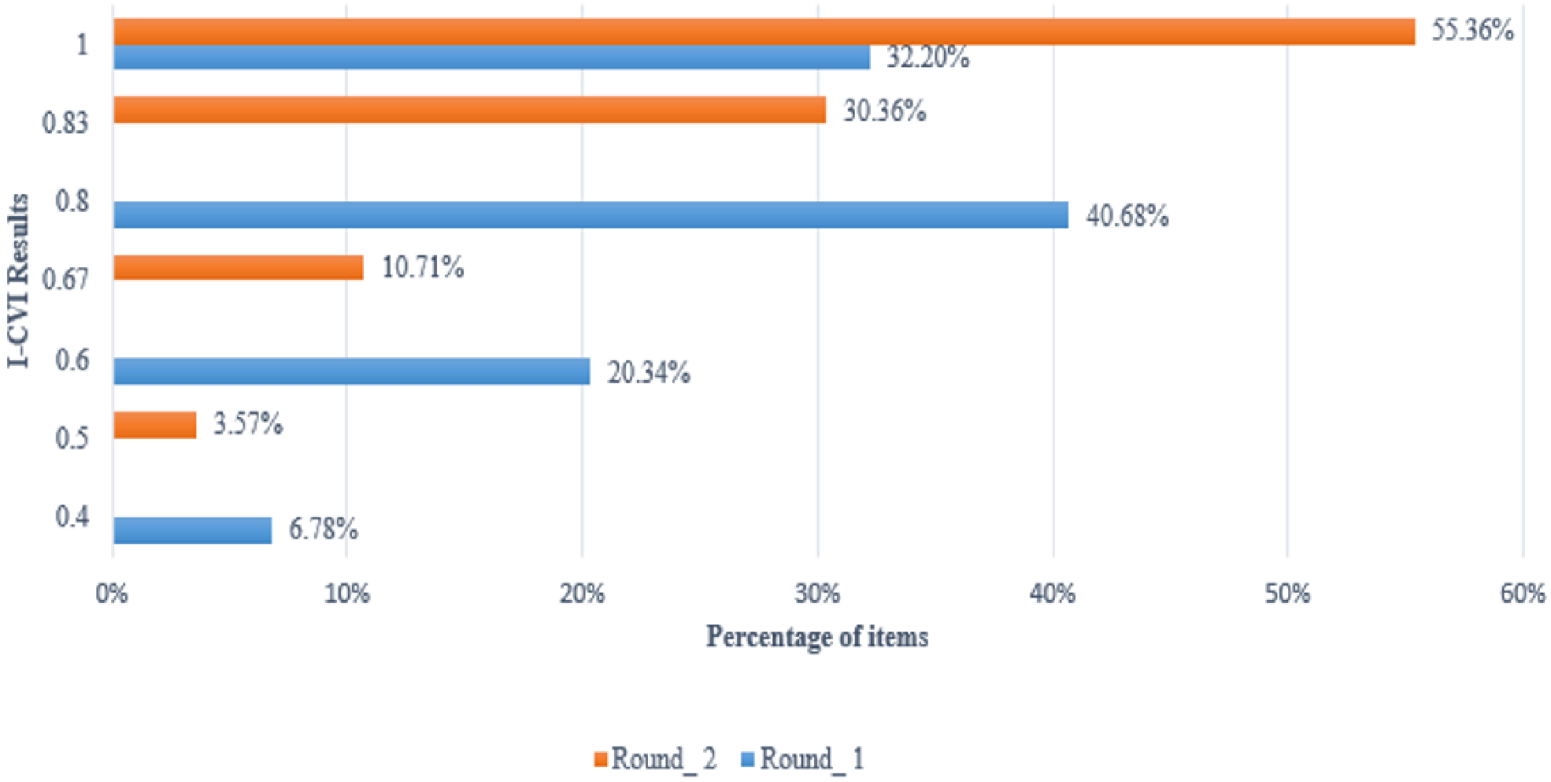
Results of I-CVI within Content Validity rounds.

In round 1, consensus (I-CVI > =1.00) was reached by the overall panel for 19 of the 59 items (32.20%). An I-CVI of 0.80 was attained for 24 items (40.68%) and 0.60 for 12 items (20.34%). In addition, an I-CVI of 0.40 was attained for only 4 of 59 items (6.78%). Figure 4 depicts the number of items in the I-CVI results. From our interpretation of the answers, the experts suggested that more refinement of the description was required for some items. The need for these refinements could have been avoided if the multi-tenancy concept was included.

In round 2, the Id of each item was redefined to reflect the resulting list of items from round 1. Table 12 also displays the I-CVIs obtained from round 2. The 31, 17, 6, and 2 of 56 items obtained I-CVIs of 1.00, 0.83, 0.67, and 0.5 respectively. In this round, all items that did not meet the minimum I-CVI of 0.78 were removed.

However, more experts were involved in round 2 than in round 1 (considering the fact that the larger the set of experts, the harder it is to reach consensus), a significant improvement of the I-CVIs results was produced in round 2. Figure 4 compares the I-CVIs of both rounds. The scores in round 1 varied from 0.4, 0.6, 0.8, and 1.00 to 0.5, 0.67, 0.83, and 1.00 in round 2. Furthermore, a significant increase in the percentage of items obtaining an I-CVI of 1.00 in round 2 was observed.

Furthermore, the kappa coefficient values in Table 12 show that 4 items, 12 items, and 43 items in round 1 received poor, fair, and excellent agreement respectively. Conversely, 2 items, 6 items, and 48 items in round 2 received poor, good, and excellent agreement respectively. Noticeably, all items with poor agreement also have poor I-CVI values.

**Figure 5:**
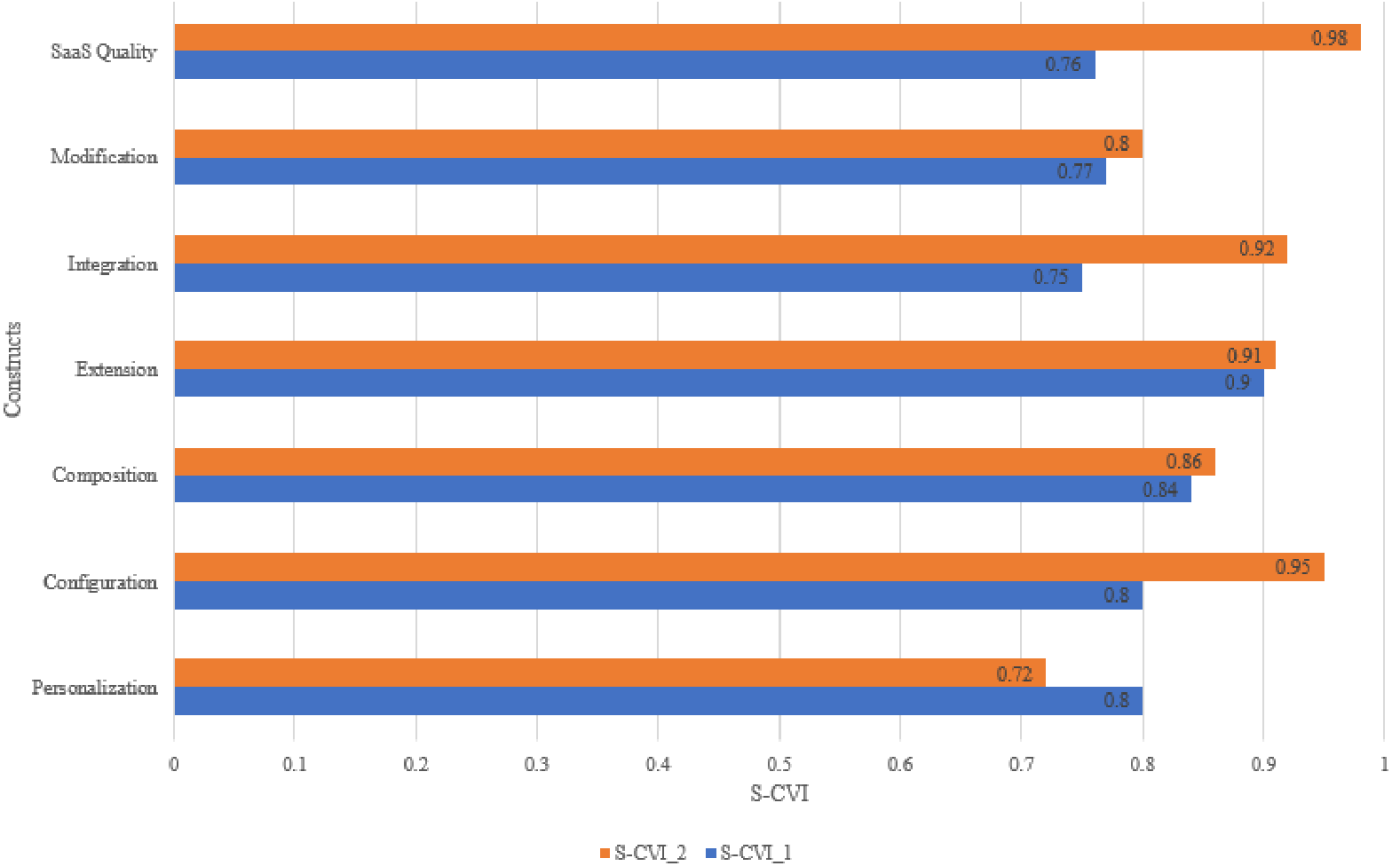
Results of S-CVI within content validity rounds.

Based on the S-CVI results in Table 12, most of the constructs attained an acceptable S-CVI in both rounds, except for the Personalization S-CVI in round 2 that was 0.72. Figure 5 shows that all S-CVI values were improved in round 2, except for the S-CVI value for Personalization that dropped from 0.8 to 0.72. The decision to delete the Personalization construct and all of its associated items was taken for the following reasons:

1. Comments from experts of both rounds indicated different interpretations; some of them thought of this construct as an alternative name for “customization, whereas others did not associate it with customization.
2. The S-CVI of 0.72 in round 2 did not meet the S-CVI criteria (> = 0.8).
3. Five of 8 items associated with this construct did not meet the I-CVI criteria (>= 0.78) in round 2.

Moreover, the S-CVIs of the Composition (0.86) and Modification (0.80) constructs in round 2 improved to 0.95 and 0.86, respectively, after removing their associated items that breached the I-CVI criteria. Detailed calculations of the I-CVIs, S-CVIs, and Kappa for rounds 1 and 2 are presented in Tables in the supplementary documents: Appendices D and E. Because of the satisfactory level of consensus indicated by the I-CVI and S-CVI scores after round 2, no further rounds were necessary.

In its final version, the software customization model for SaaS quality consisted of 45 items, grouped into six constructs: 8 items for Configuration, 4 items for Composition, 6 items for Extension, 9 items for Integration, 5 items for Modification, and 13 items for SaaS quality, as illustrated in Table 13.

**Table 13:** The development of model in each version.

**Construct**	**Original**	** Items deleted**		**Total items deleted**	**Final version**
		**Round 1**	**Round 2**		
**Personalization**	8	0	8	8	**0**
**Configuration**	9	1	0	1	**8**
**Composition**	5	0	1	1	**4**
**Extension**	6	0	0	0	**6**
**Integration**	9	0	0	0	**9**
**Modification**	9	2	2	4	**5**
**SaaS quality**	13	0	0	0	**13**
**Total**	**59**	**3**	**11**	**14**	**45**

### Internal consistency reliability test

Based on the results of the two content validity evaluation rounds, version 3 of the model was further tested regarding its internal consistency reliability using a five-point Likert scale ranging from 1 (strongly disagree) to 5 (strongly agree). In this study, selected profiles, including gender, ages, and familiarity with SaaS applications, were reported. A sample of 34 software engineering researchers completed the survey. Most of the respondents were male (*n* = 32, 94.12%). The age of the majority of respondents (55.88%) was between 31 and 40 years (*n* = 19), followed by 23.53% and 20.59% for 21–30 (*n* = 8) and over 40 (*n* = 7), respectively. The majority of respondents had an excellent knowledge of SaaS applications (*n* = 32, 94.12%) and only 5.88% (*n* = 2) were somewhat familiar with it.

The Cronbachs alpha for each construct, corrected item-total correlation, and Cronbachs alpha coefficients if the item was deleted are summarized in Table 14. Reliability analysis showed reasonable internal consistency. The computed values of Cronbachs alpha for the Configuration (*n* = 8), Composition (*n* = 4), Extension (*n* = 6), Integration (*n* = 9), and Modification (*n* = 5) constructs, as well as SaaS quality (*n* = 13) were 0.734, 0.709, 0.764, 0.814, 0.848, and 0.871, respectively. The corrected item-total correlation coefficients for Configuration items ranged from 0.301 (Con 6) to 0.522 (Con 5); Composition items ranged from 0.476 (Com 2) to 0.544 (Com 3); Extension items ranged from 0.382 (Ext 2) to 0.661 (Ext 1); Integration items ranged from 0.249 (Int 3) to 0.71 (Int 2); Modification items ranged from 0.532 (Mod 1) to 0.812 (Mod 3); and SaaS quality items ranged from 0.437 (QA 7) to 0.64 (QA 1).

**Table 14:** Reliability test results of validated model.

**Construct**	**Item**	**Cronbach’s Alpha if item deleted**	**Corrected item-total correlation**	**Construct**	**Item**	**Cronbach’s Alpha if item deleted**	**Corrected item-total correlation**
**Configuration (0.734)^a^**	Con 1	0.702	0.455	**Integration (0.814)**	Int 1	0.813	0.339
	Con 2	0.702	0.451		Int 2	0.766	0.71
	Con 3	0.716	0.381		Int 3	0.826	0.249^b^
	Con 4	0.706	0.439		Int 4	0.799	0.484
	Con 5	0.691	0.522		Int 5	0.772	0.708
	Con 6	0.731	0.301		Int 6	0.81	0.426
	Con 7	0.697	0.476		Int 7	0.783	0.608
	Con 8	0.711	0.414		Int 8	0.791	0.557
**Composition (0.709)**	Com 1	0.657	0.501		Int 9	0.788	0.572
	Com 2	0.659	0.476	**SaaS Quality (0.871)**	QA 1	0.858	0.64
	Com 3	0.632	0.544		QA 2	0.856	0.627
	Com 4	0.641	0.513		QA 3	0.861	0.545
**Extension (0.764)**	Ext 1	0.684	0.661		QA 4	0.861	0.57
	Ext 2	0.758	0.382		QA 5	0.86	0.581
	Ext 3	0.723	0.532		QA 6	0.866	0.47
	Ext 4	0.724	0.527		QA 7	0.869	0.437
	Ext 5	0.715	0.557		QA 8	0.864	0.505
	Ext 6	0.754	0.401		QA 9	0.86	0.57
**Modification (0.848)**	Mod 1	0.843	0.532		QA 10	0.857	0.629
	Mod 2	0.823	0.633		QA 11	0.866	0.507
	Mod 3	0.771	0.812		QA 12	0.858	0.619
	Mod 4	0.804	0.715		QA 13	0.864	0.493
	Mod 5	0.826	0.621				

**Notes.**

a Value between brackets is Cronbach’s Alpha results for the construct.

b Item with red colour is deleted based on Cronbach’s Alpha results if item deleted.

Table 14 also indicates that none of the items significantly reduced the value of the alpha coefficient if they were removed from the construct, except for Int 3 (in this case, the value increased from 0.814 to 0.826). Moreover, Int 3 had the lowest item-total correlation value (0.249), indicating that it did not measure the same construct as the other items. The resulting values indicate that the model has high reliability.

## Discussion

From the initial development of the software customization model for SaaS quality published in (Ali et al., 2019), we realized that the concept should be refined. The concept was initially defined based on 46 customization practices and 13 quality attributes in the SaaS multi-tenant context. Each customization practice was assigned to one of the customization approaches (8, 9, 5, 6, 9, and 9 items for Personalization, Configuration, Composition, Extension, Integration, and Modification, respectively).

To refine the model, a rigorous methodology, composed of an iterative content validity evaluation process and a reliability test, was followed. During the two content validity rounds, answers and comments were suggested by experts to further refine the language used and explicitly declare the multi-tenancy concept in the items. Consequently, the I-CVIs and S-CVIs results varied between rounds 1 and 2.

In round 1, the items that breached the I-CVI criteria were re-written. Moreover, a reduction in the number of items (from 59 to 56) was achieved. Similarly, version 3, consisting of 45 items, was created after round 2. A total of 11 items were deleted in this round; 2, 1, and 8 items were deleted from the Modification, Composition, and Personalization constructs, respectively. Although 3 of 8 items of the Personalization construct did not breach the I-CVI criteria, they were deleted due to the removal of the Personalization construct that did not meet the S-CVI criteria.

Several experts had conflicting opinions regarding the Personalization construct. One opinion was that Personalization is a synonym word for customization, hence, all approaches proposed in this study should be considered Personalization approaches. The second point of view was that it is completely different from customization as it does not involve any customer action, which is essential for customization. The authors of this study agreed with the second opinion. The initial inclusion of Personalization as an approach to Customization in this study was due to the ultimate purpose of both mechanisms to meet the unique requirements of the customer by adapting the application to their needs.

The results of rounds 1 and 2 indicated considerable discrepancy in the numbers of items deleted and revised. The number of items deleted in round 1 (3) was lower than that in round 2 (11). By contrast, the number of items revised in round 1 (21 items) was higher than that in round 2 (0). This result, however, is expected as the objective of the first round was to revise the items that did not meet the I-CVI criteria rather than delete them. The purpose of round 2, however, was to remove any item that did not meet the criteria. This strategy, therefore, allowed most of the items to be assessed twice. Moreover, with this strategy, stability in the response of experts was also achieved with the recommended minimum number of rounds (two rounds) (Landeta, 2006), overcoming the limitations of iteration structure methods (e.g., the Delphi method), which does not specify any number of rounds (Keeney, Hasson & McKenna, 2001).

**Figure 6:**
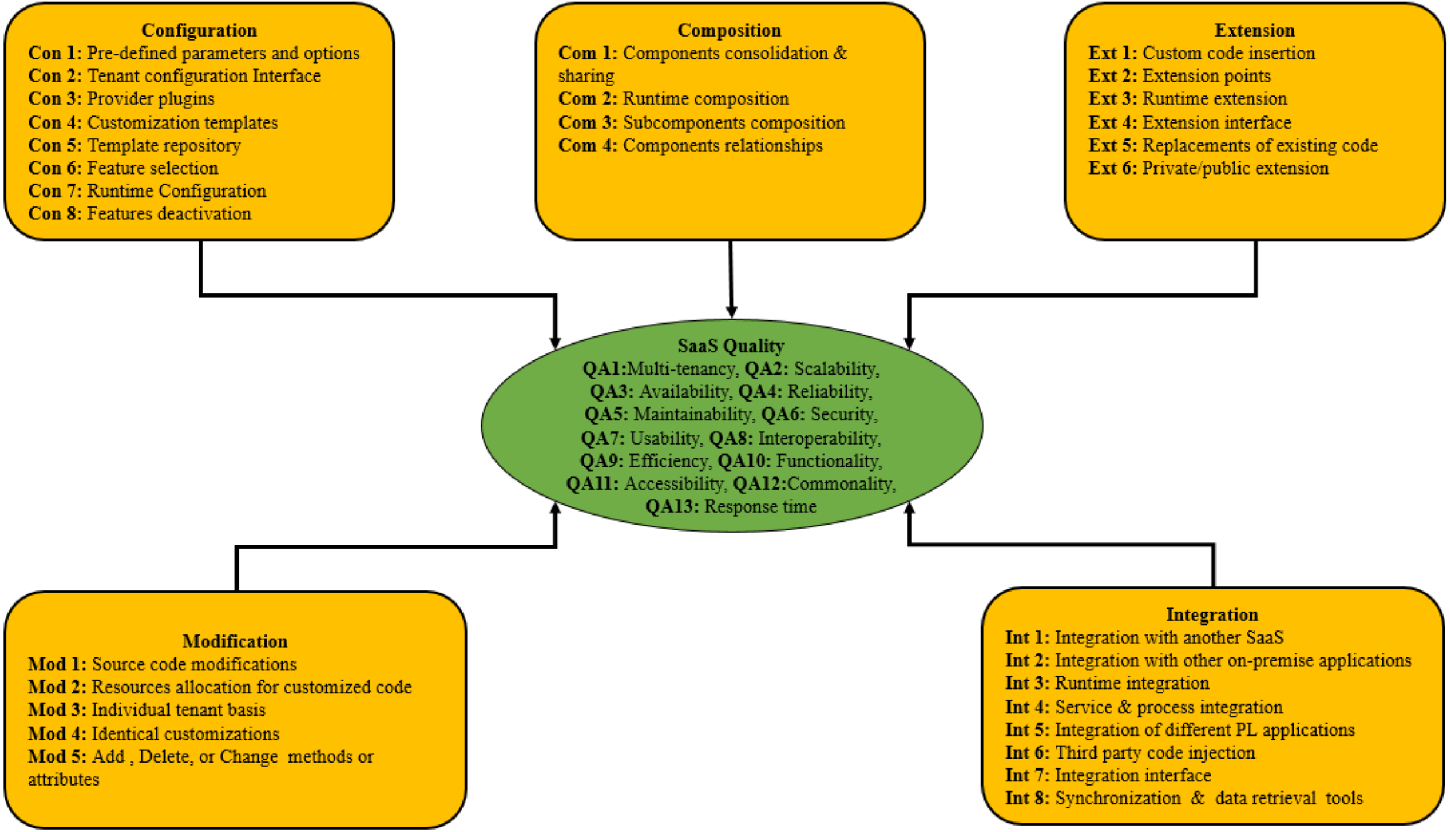
Final proposed software customization model for SaaS quality.

The consensus for content validity was reached and an additional round was included to test the internal consistency reliability of the model items and constructs. In this round, software engineer researchers were asked to reassess the items and evaluate them using a 5- point Likert-type scale. The reliability, found by using Cronbachs alpha, is proof of items consistently representing the constructs. In this test, only one item was deleted to increase the value of Cronbachs alpha. At the end of this round, all constructs and items achieved the required values of reliability and validity. The final version of the proposed model is shown in Fig. 6.

## Threats to Validity

Three major limitations emerge. These include sample size, selection bias, and modification bias.

### Sample size

The experts involved in the content validation rounds numbered 5 in the first round and 6 in the second round. Although this sample is fairly small for the iterative method, smaller numbers are considered sufficient for homogeneous samples (Hong et al., 2019). Moreover, when using the content validity index (CVI) method, 5 experts are considered sufficient to validate the model (Zamanzadeh et al., 2015). Because our samples were relatively homogeneous (academicians) in terms of participants, expertise, 3 to 10 experts are sufficient for the adopted CVI analysis method, and more than 10 experts would be unnecessary (Polit & Beck, 2006). Accordingly, the number of experts used in this study should be considered acceptable.

Another issue with our sample size is the imbalance in the numbers of experts in rounds 1 and 2. The increased number of experts from 5 to 6 in round 2 was because the group of experts invited to participate in the second round was larger. Although the required threshold value for consensus decreases as the number of experts increases, it is harder to achieve consensus with larger numbers. As such, the increase from 5 to 6 in round 2 did not skew the results of this study. Additionally, it is not required to have a consistent number of participants in all rounds of a study; for instance, Cadorin et al. (2017) had 10 participants in the first round and 8 in the subsequent rounds of their study.

### Selection bias

The selection of experts is essential for obtaining valid and reliable results. Compared to random selection methods, our purposive sampling of experts may have led to selection bias. In our study, four possible issues related to selection bias were identified:

1. *Self-selection bias:* This concern was mitigated by identifying and approaching the most suitable experts for our study via an extensive systematic mapping study (Ali et al., 2019).
2. *Homogeneous sample:* The diversity of experts strengthens the statistical power and the generalizability of results; however, a homogeneous sample in the studies that used the iterative method is acceptable to facilitate group decision-making process (Skulmoski, Hartman & Krahn, 2007; Logue & Effken, 2013).
3. *Bias of superior individuals*: Experts were approached based on their published papers (81 papers) that were most related to this study, and every paper had more than one author. Therefore, there is a possibility that the experts who participated in this study are from the same organization or university, and in such a case, there is a real possibility that the ideas and opinions of one expert will be influenced by more dominant experts in the same organization (Mubarak et al., 2019; Fletcher & Marchildon, 2014). Accordingly, the experts opinions were collected anonymously via e-mail without being affected or pressured by other individuals (Mubarak et al., 2019; Halim et al., 2017; Stevens et al., 2006).
4. *Different experts in each round:* Another possible limitation is having different expert panels in each round, which is not common in iterative methods (Stevens et al., 2006; Parratt et al., 2016). Although having the same experts in the initial round who continued to participate in all rounds of a study provides the opportunity for the experts to alter their opinions in successive rounds based on the results of previous rounds to achieve consensus (Stevens et al., 2006), the results may be influenced by forced consensus through conformity and diverse opinions being relinquished (Parratt et al., 2016). Considering this fact, having different experts participate in each round may arguably improve the results of a study (Parratt et al., 2016). It is worth noting that the survey for round 2 was sent to the same experts who were involved in the initial round and none responded within the time limit, leading to new respondents being selected for the second round. In addition, as participation in our study was voluntary, those who participated in round 1 may not have had the time or inclination to continue.

### Modification bias

The model manipulation applied in this study resulted in the number of constructs being reduced from 7 to 6 by the removal of the Personalization construct and associated items that did not attain an acceptable CVIs value. Although this modification to the model may have added a certain level of bias, the deletion of the Personalization construct is indirectly supported by the findings of SMS, where Personalization received the lowest consideration of all customization solutions proposed for SaaS applications. Furthermore, we followed the strategy of revising the items that did not meet the I-CVI criteria rather than deleting them in round 1, leaving the deletion of the invalid item(s)/construct to the subsequent rounds. This strategy provided the opportunity for most of the items to be assessed at least twice. Eventually, the deletion of the Personalization construct and other items was deemed necessary for the study on grounds supported in the literature and by experts’ comments.

## Conclusions

The comprehension of the generic customization approaches and practices in the SaaS multi-tenant context and the identification of the key quality attributes of SaaS applications associated with customization is an opportunity to increase the understanding of SaaS customization, creating further discussions of the subject. The purpose of this study was, therefore, to develop a software customization model for SaaS quality to identify possible customization approaches, practices, and quality attributes in the SaaS Multi-Tenant context. In addition, this study can be considered the first one, to the best of the authors’ knowledge, to develop a theoretical, validated, and reliable software customization model for SaaS quality. To evaluate this model, an iterative method was used to conceptualize it, assess its content validity, and evaluate its reliability.

A preliminary version of this model, composed of seven constructs (six customization approaches and SaaS quality) and 59 items (46 SaaS customization practices and 13 SaaS quality attributes), was used. After the completion of two rounds of content validity evaluation, one construct and 14 items were removed. To improve the reliability of the validated model, round 3 was executed and all constructs achieved the required Cronbachs alpha value. Furthermore, the removal of only one item significantly reduced the Cronbachs alpha value. The final version of the model consisted of six constructs and 44 items. These six constructs and their associated items are as follows: 1) Configuration (eight items), 2) Composition (four items), 3) Extension (six items), 4) Integration (8 items), 5) Modification (five items), and 6) SaaS quality (13 items).

However, the model that was iteratively validated offers some certainty of construct validity, our ongoing research is to evaluate its construct validity and reliability with a larger sample of SaaS implementation team members, based on the industry environment. In addition, this study is restricted to the quality attributes of SaaS applications from a systematic mapping study (Ali et al., 2019). However, this study does not claim that only these SaaS quality attributes are associated with customization. Future studies could also be conducted to expand the model to include many other quality attributes of SaaS applications, especially SaaS attributes related to the affordability quality attribute (e.g., resource cost and maintenance costs). The key contribution of this study is that it advances existing knowledge on SaaS customization and quality by the development and validation of a software customization model. It also enhances the potential to analyze empirically the impact of software customization on SaaS quality from a software professionals perspectives. This study can be used as a source of qualitative and quantitative data for further investigation into the statistical linkage between software customization and SaaS quality. The findings of these future investigations will prompt evaluators, testers, and developers of SaaS applications to resolve quality-related issues before any customization is introduced.

##  Supplemental Information

10.7717/peerj-cs.294/supp-1 Supplemental Information 1 Questionnaires of versions 1, 2, and 3 of the model We conducted two rounds of content validity between February 2019 and June 2019 starting with version 1 309 of the model produced from the conceptualization phase. It was revised after each round, therefore, generating 310 versions 2 and 3.

10.7717/peerj-cs.294/supp-2 Supplemental Information 2 Detailed calculations of the I-CVIs, S-CVIs, and Kappa for rounds 1

10.7717/peerj-cs.294/supp-3 Supplemental Information 3 Detailed calculations of the I-CVIs, S-CVIs, and Kappa for rounds

## Additional Information and Declarations

### Data Availability

The following information was supplied regarding data availability: The raw measurements are available in the Supplementary Files.
