# Supplementary material for: Development of a valid and reliable software customization model for SaaS quality through iterative method: perspectives from academia
Source: PeerJ Comput Sci. 2020 Sep 21;6:e294. doi: 10.7717/peerj-cs.294 (PMC7924678; doi:10.7717/peerj-cs.294)
Supplement: Supplemental Information 1 — We conducted two rounds of content validity between February 2019 and June 2019 starting with version 1 309 of the model produced from the conceptualization phase. It was revised after each round, therefore, generating 310 versions 2 and 3. [file peerj-cs-06-294-s001.pdf]

Appendix A

Content Validity Questionnaire (Round 1)

SaaS Personalization

Personalization refers to techniques and solutions that provide transparent customization without a need for the users to be informed and it is initiated by the application.

Please indicate to what relevancy you feel these statements represent the **personalization** approach in the SaaS Multi-Tenant context.

	Questions	Not relevant	Somewhat relevant	Relevant	Very relevant	Comment *(if any)*
1.	Personalization involves amassing data sets correlating to individual user.	○	○	○	○	
2.	Personalization seeks to integrate users' autonomous selections within SaaS services.	○	○	○	○	
3.	Personalization involves assembling data on clients' activities regarding individual services.	○	○	○	○	
4.	Personalization concerns sets of potential services which can be presented to users.	○	○	○	○	
5.	Personalization within SaaS entails complex methods for recommending suitable services according to users' established partialities, user profiles, data usage, and service directories.	○	○	○	○	
6.	SaaS-based personalization considers the meaning (semantics) of data, in addition to formulating suggestions and recommendations.	○	○	○	○	
7.	SaaS-based personalization uses runtime behavior adaptation facilities that can creatively modify the behavior of SaaS applications in accordance with the context of their performance.	○	○	○	○	
8.	Information sources for SaaS-based personalization can originate from a specific user or from tenant communities.	○	○	○	○	

SaaS Configuration

Configuration refers to techniques and solutions that offer a pre-defined setting for the alteration of application functions within the pre-defined scope.

Please indicate to what relevancy you feel these statements represent the **configuration** approach in the SaaS Multi-Tenant context.

Questions	Not relevant	Somewhat relevant	Relevant	Very relevant	Comment *(if any)*
1. Configuration typically maintains diversity by establishing pre-defined parameters and options.	○	○	○	○	
2. Configuration can also be operated in a standalone way by employing techniques to modify the functions of applications within established limits. For example, in relation to the wizards provided by the customization of UI utilities.	○	○	○	○	
3. SaaS service providers have developed and captured sets of services and plugins, from which tenants can make selections and perform configurations.	○	○	○	○	
4. Tenants can create customization based on templates.	○	○	○	○	
5. Tenants can select their own workflow templates and items relating to SaaS application templates from the template repository.	○	○	○	○	
6. A set of components are provided in the application template which facilitates a variety of tenant needs. By making a choice from the relevant component set, tenants can personalize each customization point.	○	○	○	○	
7. When a tenant wishes to subscribe to the SaaS application, the capabilities of each feature within the system are analyzed to determine whether they ought to be assimilated within the application.	○	○	○	○	
8. Configuration can manage incongruities by permitting the client to establish set pre-defined parameters and options within the context of the runtime.	○	○	○	○	
9. The configuration of the SaaS application involves disabling or excluding some features of the application.	○	○	○	○	

SaaS Composition

Composition refers to techniques and solutions that bring together a distinct collection of pre-defined application components that jointly amount to a custom solution.

Please indicate to what relevancy you feel these statements represent the **composition** approach in the SaaS Multi-Tenant context.

Questions	Not relevant	Somewhat relevant	Relevant	Very relevant	Comment *(if any)*
1. The multiple interacting components of the SaaS application are consolidated, and new application components can be shared between multiple SaaS tenants and end users.	○	○	○	○	
2. Composing different collaboration components is done according to the runtime of the SaaS application.	○	○	○	○	
3. The composition of SaaS components takes into account the subset of components.	○	○	○	○	
4. The composition approach supports the decomposition of SaaS components.	○	○	○	○	
5. Performing the composition of SaaS application components considers the relationships and dependencies between these components.	○	○	○	○	

SaaS Extension

Extension refers to techniques and solutions that that stretch the functionality of the application by implanting the custom code in pre-defined places of application's code.

Please indicate to what relevancy you feel these statements represent the **extension** approach in the SaaS Multi-Tenant context.

Questions	Not relevant	Somewhat relevant	Relevant	Very relevant	Comment *(if any)*
1. The SaaS application is extended by adding a custom code to extend the application through custom functionality.	○	○	○	○	

	Questions	Not relevant	Somewhat relevant	Relevant	Very relevant	Comment *(if any)*
2.	The SaaS application provides a set of extension points which permit a customized service to be plugged in at virtually points in the application.	○	○	○	○	
3.	Extending an existing object can happen at SaaS application runtime.	○	○	○	○	
4.	The SaaS service provider supplies an open platform and an API, which allows developers to inject custom codes into business object layers.	○	○	○	○	
5.	These extension points can either be replacements for existing objects or extensions to them.	○	○	○	○	
6.	An extension may be private to an individual tenant or shared by multiple tenants.	○	○	○	○	

SaaS Integration

Integration refers to techniques and solutions that Implement third-party components designed to work with the application.

Please indicate to what relevancy you feel these statements represent the **integration** approach in the SaaS Multi-Tenant context.

	Questions	Not relevant	Somewhat relevant	Relevant	Very relevant	Comment *(if any)*
1.	SaaS application functionality can be expanded through the addition of extra services via external SaaS providers.	○	○	○	○	
2.	Most SaaS service customers assume that the SaaS application will be easy to amalgamate with their existing in-house systems	○	○	○	○	
3.	The integration of SaaS applications with external systems related to non-functional elements, such as security controls, should be facilitated by SaaS architecture.	○	○	○	○	
4.	Integration encompasses elements which safeguard an	○	○	○	○	

	Questions	Not relevant	Somewhat relevant	Relevant	Very relevant	Comment *(if any)*
	unbroken stream of integration at both design time and runtime.					
5.	Integration platforms incorporate both service frameworks, through which services and can be assimilated, and process frameworks, through which business processes can be performed.	○	○	○	○	
6.	Additional services from third-party SaaS providers employ different programming languages running in different contexts.	○	○	○	○	
7.	Coding or scripting is utilized to incorporate services into applications.	○	○	○	○	
8.	Incorporating services into applications requires configuration or setup.	○	○	○	○	
9.	Synchronization toolkits and data retrieval mechanisms are created to respond to the demands posed by integration.	○	○	○	○	

SaaS Modification

Modification refers to techniques and solutions that alter the application design and other functional requirements of the application by way of alterations implemented on the source code.

Please indicate to what relevancy you feel these statements represent the **modification approach** in the SaaS Multi-Tenant context.

	Questions	Not relevant	Somewhat relevant	Relevant	Very relevant	Comment *(if any)*
1.	The generation of functionality requires source code modifications to be made as part of the customization process.	○	○	○	○	
2.	Overseeing different incarnations of software codes necessitates the effective distribution of infrastructure and assets.	○	○	○	○	
3.	SaaS vendors must manage all elements of	○	○	○	○	

	Questions	Not relevant	Somewhat relevant	Relevant	Very relevant	Comment *(if any)*
	customization codes on an individual tenant basis.					
4.	SaaS vendors can alter application codes in cases where the definition of configuration and customization by large tenant groups justifies or requires this.	○	○	○	○	
5.	Code customization changes are implemented at the runtime of the SaaS application.	○	○	○	○	
6.	Runtime code changes have to consider the dependency relationship between different functions, whereby one function can depend on several functions yet can simultaneously be depended on by several others.	○	○	○	○	
7.	Namespaces, inheritance, and polymorphism are used to implement source code customizations.	○	○	○	○	
8.	Source code modifications are instituted by adding new methods or attributes, or by changing the object's current implementation methods.	○	○	○	○	
9.	Source code modifications involve the deletion of custom objects, methods, or attributes.	○	○	○	○	

Part 3: SaaS Quality

Based on the definition provided for each quality attribute, please indicate to what relevancy you feel these statements represent the **quality attributes** of SaaS application that play an important role in customization.

	Questions	Not relevant	Somewhat relevant	Relevant	Very relevant	Comment *(if any)*
1.	**Multi-tenancy:** SaaS services can support instances of simultaneous access by multiple users for multiple tenants.	○	○	○	○	
2.	**Scalability:** SaaS providers can manage growth or decline in the level of services.	○	○	○	○	
3.	**Availability:** SaaS services can function within a specific time to satisfy users' needs.	○	○	○	○	
4.	**Reliability:** SaaS services maintain operations and functioning without failure within a given time period.	○	○	○	○	

	Questions	Not relevant	Somewhat relevant	Relevant	Very relevant	Comment *(if any)*
5.	**Maintainability:** SaaS providers can repair services to keep them in good working order.	○	○	○	○	
6.	**Security:** SaaS providers control service data and access to the services.	○	○	○	○	
7.	**Usability:** A service can be perceived as useful and accessible by clients when used according to its intended application.	○	○	○	○	
8.	**Interoperability:** SaaS service can easily interact with other services.	○	○	○	○	
9.	**Efficiency**: SaaS services effectively utilize resources to perform their functions.	○	○	○	○	
10.	**Functionality:** SaaS service features are extensive/inclusive.	○	○	○	○	
11.	**Accessibility:** The service is suitable for use by users with disabilities.	○	○	○	○	
12.	**Commonality:** SaaS services possess common features and are amenable to reuse by multiple users.	○	○	○	○	
13.	**Response time:** There is defined time limit which is adhered to between a service request and a service response.	○	○	○	○	

Comments and Suggestions

If there are any other statements, or further comments regarding the customization approaches or the quality attributes of SaaS applications that you think is needed and have not reflected in this survey, please add your remarks in the space provided below.

Thank you for your time

Appendix B

Content Validity Questionnaire (Round 2)

SaaS Personalization

Personalization refers to techniques and solutions that provide transparent customization initiated by the application without the need to inform the users.

Please indicate to what relevancy you feel these statements represent the **personalization** approach in the SaaS Multi-Tenant context.

	Questions	Not relevant	Somewhat relevant	Quite Relevant	Very relevant	Comment *(if any)*
1.	Personalization involves gathering data sets correlating to individual tenant or group of tenants.	○	○	○	○	
2.	Personalization seeks to learn accurate services based on the tenant's current preferences, and other tenants' shared preferences	○	○	○	○	
3.	Personalization involves collecting data on tenant's activities and take advantage of other tenants behavioral activities	○	○	○	○	
4.	Personalization uses a set of potential services offered by pre-structured templates of multiple SaaS providers.	○	○	○	○	
5.	Personalization within SaaS entails recommendation mechanism for proposing suitable services according to users' preferences, user profiles, data usage, and service directories.	○	○	○	○	
6.	SaaS-based personalization considers the meaning (semantics) of data of tenants and tenants' community	○	○	○	○	
7.	SaaS-based personalization uses runtime behavior adaptation facilities that can creatively modify the behavior of SaaS applications in accordance with the context of their performance.	○	○	○	○	
8.	Information sources for SaaS-based personalization can originate from a tenant or tenant's communities.	○	○	○	○	

SaaS Configuration

Configuration refers to techniques and solutions that offer a pre-defined setting for the alteration of application functions within the pre-defined scope.

Please indicate to what relevancy you feel these statements represent the **configuration** approach in the SaaS Multi-Tenant context.

	Questions	Not relevant	Somewhat relevant	Relevant	Quite relevant	Very relevant	Comment *(if any)*
1.	Configuration typically maintains diversity by establishing pre-defined parameters, options, and components, and treats each tenant individually.	○	○	○	○		
2.	Each tenant can configure the application in a standalone way by employing techniques to modify the functions of applications within established limits.	○	○	○	○		
3.	SaaS providers have to develop and capture sets of services and plugins, from which tenants can make selections and perform configurations.	○	○	○	○		
4.	Tenants can create customization based on templates.	○	○	○	○		
5.	Tenants can select their desired workflow templates and items relating to SaaS application templates from the template repository.	○	○	○	○		
6.	When a tenant wishes to subscribe to the SaaS application, the capabilities of each feature within the system are analyzed to determine whether they ought to be assimilated within the application.	○	○	○	○		
7.	All Configurations established by the tenants have to be within the context of the runtime of the application.	○	○	○	○		
8.	An option of disabling or excluding some features of the SaaS application should be provided with the isolation effect across the tenants.	○	○	○	○		

SaaS Composition

Composition refers to techniques and solutions that bring together a distinct collection of pre-defined application components that jointly amount to a custom solution.

Please indicate to what relevancy you feel these statements represent the **composition** approach in the SaaS Multi-Tenant context.

Questions		Not relevant	Somewhat relevant	Relevant	Quite relevant	Very relevant	Comment *(if any)*
1.	The multiple interacting components of the SaaS application are consolidated, and new application components can be shared between multiple SaaS tenants and end users.	◌	◌	◌	◌		
2.	Composing different collaboration components is done according to the runtime of the SaaS application.	◌	◌	◌	◌		
3.	The composition of components takes into account the subcomponents of the core one.	◌	◌	◌	◌		
4.	The composition approach supports the simplification of consolidated SaaS components.	◌	◌	◌	◌		
5.	Performing the composition of SaaS application components considers the relationships and dependencies between these components.	◌	◌	◌	◌		

SaaS Extension

Extension refers to techniques and solutions that expand the functionality of the application by inserting the custom code in pre-defined places of application's code.

Please indicate to what relevancy you feel these statements represent the **extension** approach in the SaaS Multi-Tenant context.

Questions		Not relevant	Somewhat relevant	Relevant	Quite relevant	Very relevant	Comment *(if any)*
1.	The SaaS application is extended by adding custom code to extend the application through custom functionality.	◌	◌	◌	◌		
2.	The SaaS application provides a set of extension points which permit a customized service to be plugged in at virtual points in the application.	◌	◌	◌	◌		
3.	Injecting custom code into SaaS application has to be	◌	◌	◌	◌		

	Questions	Not relevant	Somewhat relevant	Relevant	Quite relevant	Very relevant	Comment *(if any)*
	supported at the run time of the application.						
4.	The SaaS service provider supplies an open platform and an API, which allows developers to inject custom codes into business object layers.	○	○	○	○		
5.	These injected codes can either be replacements for existing objects or extensions to them.	○	○	○	○		
6.	An extension may be private to an individual tenant or shared by multiple tenants.	○	○	○	○		

SaaS Integration

Integration refers to techniques and solutions that implement third-party components designed to work with the application.

Please indicate to what relevancy you feel these statements represent the **integration** approach in the SaaS Multi-Tenant context.

	Questions	Not relevant	Somewhat relevant	Relevant	Quite relevant	Very relevant	Comment *(if any)*
1.	SaaS application functionality can be expanded through the addition of extra services via external SaaS providers.	○	○	○	○		
2.	SaaS service customers assume that the SaaS application will be easy to amalgamate with their existing in-house systems	○	○	○	○		
3.	The integration of SaaS applications with external systems related to non-functional elements, such as security controls, should be facilitated by SaaS architecture.	○	○	○	○		
4.	Integration encompasses aspects which ensure a smooth flow at both design time and runtime.	○	○	○	○		
5.	Integration platforms incorporate both service framework, through which services can be assimilated, and process framework, through which business processes can be executed.	○	○	○	○		

	Questions	Not relevant	Somewhat relevant	Relevant	Quite relevant	Very relevant	Comment *(if any)*
6.	Additional services from third-party SaaS providers employ different programming languages running in different environments.	◌	◌	◌	◌		
7.	Coding or scripting is utilized to incorporate external services into SaaS application.	◌	◌	◌	◌		
8.	Incorporating services into SaaS application requires an integration interface in the form of configuration or setup.	◌	◌	◌	◌		
9.	Synchronization toolkits and data retrieval mechanisms are created to respond to the demands posed by integration.	◌	◌	◌	◌		

SaaS Modification

Modification refers to techniques and solutions that alter the application design and other functional requirements of the application by means of alterations implemented to the source code.

Please indicate to what relevancy you feel these statements represent the **modification approach** in the SaaS Multi-Tenant context.

	Questions	Not relevant	Somewhat relevant	Relevant	Quite relevant	Very relevant	Comment *(if any)*
1.	Source code modifications are made to SaaS application to generate a new functionality without changing a shared code base.	◌	◌	◌	◌		
2.	The code modification must take resources allocation for customized code into account, ensuring operational cost-efficiency in terms of maintenance costs and resource sharing among tenants.	◌	◌	◌	◌		
3.	SaaS vendors must manage all elements of customization codes on an individual tenant basis without developing many software versions for each	◌	◌	◌	◌		

	Questions	Not relevant	Somewhat relevant	Relevant	Quite relevant	Very relevant	Comment *(if any)*
	tenant.						
4.	SaaS vendors alter application codes when identical customizations are defined and justified by a considerable number of tenants.	○	○	○	○		
5.	Code customization changes are implemented at the runtime of the SaaS application.	○	○	○	○		
6.	Runtime code changes have to consider the dependency relationship between different functions, whereby one function of SaaS application can depend on several functions yet can simultaneously be depended on by several others.	○	○	○	○		
7.	Source code modifications are made by adding/deleting methods or attributes, or by changing the object's current implementation methods.	○	○	○	○		

Part 3: SaaS Quality

Based on the definition provided for each quality attribute, please indicate to what relevancy you feel these statements represent the **quality attributes** of SaaS application that play an important role in SaaS customization.

	Questions	Not relevant	Somewhat relevant	Relevant	Quite relevant	Very relevant	Comment *(if any)*
1.	**Multi-tenancy:** SaaS services can support instances of simultaneous access by multiple users for multiple tenants.	○	○	○	○		
2.	**Scalability:** SaaS providers can manage growth or decline in the level of services.	○	○	○	○		
3.	**Availability:** SaaS services can function within a specific time to satisfy users' needs.	○	○	○	○		
4.	**Reliability:** SaaS application maintains operating and functioning under given conditions without failure within a given time period.	○	○	○	○		

	Questions	Not relevant	Somewhat relevant	Relevant	Quite relevant	Very relevant	Comment *(if any)*
5.	**Maintainability:** Modifications to the application are made by SaaS provider to retain it in the condition of good repair.	○	○	○	○		
6.	**Security:** the effectiveness of SaaS provider's controls on service data, access to the services, and the physical facilities from which service are provided.	○	○	○	○		
7.	**Usability:** the ease with which SaaS application can be used to achieve tenant-specific-goal.	○	○	○	○		
8.	**Interoperability:** SaaS service can easily interact with other services from the same SaaS provider or other providers.	○	○	○	○		
9.	**Efficiency**: SaaS services effectively utilize resources to perform their functions.	○	○	○	○		
10.	**Functionality:** SaaS application provides an extensive set of features.	○	○	○	○		
11.	**Accessibility:** SaaS services are operable by users with different disabilities.	○	○	○	○		
12.	**Commonality:** SaaS services possess common features and are amenable to reuse by multiple users.	○	○	○	○		
13.	**Response time:** SaaS application adheres to a defined time limit between service request and service response.	○	○	○	○		

Comments and Suggestions

If there are any other statements, or further comments regarding the customization approaches or the quality attributes of SaaS applications that you think is needed and have not reflected in this survey, please add your remarks in the space provided below.

Appendix C

Reliability Questionnaire (Round 3)

Part 1: Demographics

Please mark your response for each of the following questions:

1- What is your gender?

- ○ Male
- ○ Female
- ○ Other

2- Which age range describes you best?

- ○ 21-30
- ○ 31-40
- ○ Over 40

3- Are you familiar with SaaS (Software as a Service) in Cloud Computing?

- ○ Yes
- ○ No
- ○ Somewhat

4- which SaaS applications are you familiar?

- ☐ Google maps and apps (mails, docs, and drive)
- ☐ Microsoft Office 365
- ☐ Salesforce CRM application
- ☐ And others

5- How many years' experience do you possess in the following areas?

	None	1-2	3-4	>4
Software engineering	○	○	○	○
Software development	○	○	○	○
Software quality	○	○	○	○
Software maintenance and operation	○	○	○	○
Software customization	○	○	○	○
Multi-Tenant SaaS	○	○	○	○

Part 2: SaaS Customization Approaches

The following statements describe the different customization approaches that may impact the quality of SaaS applications. The scales below represent opinions of equivalent weight and strength. You should select the responses which most closely correspond to your views. For each question, you must choose one scale ONLY.

SaaS Configuration

Configuration refers to techniques and solutions that offer a pre-defined setting for the alteration of application functions within the pre-defined scope.

Please indicate the extent to which, in your view, the following statements represent the **configuration** approach in the SaaS Multi-Tenant context.

	**Questions**	**Strongly Disagree**	**Disagree**	**Neither Nor**	**Agree**	**Strongly agree**
1.	Configuration typically maintains diversity by establishing pre-defined parameters, options, and components, and treats each tenant individually.	○	○	○	○	○
2.	Each tenant can configure the application in a standalone way by employing techniques to modify the functions of applications within established limits.	○	○	○	○	○
3.	SaaS providers have to develop and capture sets of services and plugins, from which tenants can make selections and perform configurations.	○	○	○	○	○
4.	Tenants can create customization based on templates.	○	○	○	○	○
5.	Tenants can select their desired workflow templates and items relating to SaaS application templates from the template repository.	○	○	○	○	○
6.	When a tenant wishes to subscribe to the SaaS application, the capabilities of each feature within the system are analyzed to determine whether they ought to be assimilated within the application.	○	○	○	○	○
7.	All Configurations established by the tenants have to be within the context of the runtime of the application.	○	○	○	○	○
8.	An option of disabling or excluding some features of the SaaS application should be provided with the isolation effect across the tenants.	○	○	○	○	○

SaaS Composition

Composition refers to techniques and solutions that bring together a distinct collection of pre-defined application components that jointly amount to a custom solution.

Please indicate the extent to which you feel the following statements represent the **composition** approach in the SaaS Multi-Tenant context.

Questions	Strongly Disagree	Disagree	Neither Nor	Agree	Strongly agree
1. The multiple interacting components of the SaaS application are consolidated, and new application components can be shared between multiple SaaS tenants and end users.	○	○	○	○	○
2. Composing different collaboration components is done according to the runtime of the SaaS application.	○	○	○	○	○
3. The composition of components takes into account the subcomponents of the core one.	○	○	○	○	○
4. Performing the composition of SaaS application components considers the relationships and dependencies between these components.	○	○	○	○	○

SaaS Extension

Extension refers to techniques and solutions that expand the functionality of the application by inserting the custom code in pre-defined places of application's code.

Please indicate the extent to which, in your view, you feel the following statements represent the **extension** approach in the SaaS Multi-Tenant context.

Questions	Strongly Disagree	Disagree	Neither Nor	Agree	Strongly agree
1. The SaaS application is extended by adding custom code to extend the application through custom functionality.	○	○	○	○	○
2. The SaaS application provides a set of extension points which permit a customized service to be plugged in at virtual points in the application.	○	○	○	○	○
3. Injecting custom code into SaaS application has to be supported at the run time of the application.	○	○	○	○	○

Questions	Strongly Disagree	Disagree	Neither Nor	Agree	Strongly agree
4. The SaaS service provider supplies an open platform and an API, which allows developers to inject custom codes into business object layers.	○	○	○	○	○
5. These injected codes can either be replacements for existing objects or extensions to them.	○	○	○	○	○
6. An extension may be private to an individual tenant or shared by multiple tenants.	○	○	○	○	○

SaaS Integration

Integration refers to techniques and solutions that implement third-party components designed to work with the application.

Please indicate the extent to which you feel the following statements represent the **integration** approach in SaaS Multi-Tenant context.

Questions	Strongly Disagree	Disagree	Neither Nor	Agree	Strongly agree
1. SaaS application functionality can be expanded through the addition of extra services via external SaaS providers.	○	○	○	○	○
2. SaaS service customers assume that the SaaS application will be easy to amalgamate with their existing in-house systems	○	○	○	○	○
3. The integration of SaaS applications with external systems related to non-functional elements, such as security controls, should be facilitated by SaaS architecture.	○	○	○	○	○
4. Integration encompasses aspects which ensure a smooth flow at both design time and runtime.	○	○	○	○	○
5. Integration platforms incorporate both service framework, through which services can be assimilated, and process framework, through which business processes can be executed.	○	○	○	○	○
6. Additional services from third-party SaaS providers employ different programming languages running in different environments.	○	○	○	○	○
7. Coding or scripting is utilized to incorporate external	○	○	○	○	○

Questions	Strongly Disagree	Disagree	Neither Nor	Agree	Strongly agree
services into SaaS application.					
8. Incorporating services into SaaS application requires an integration interface in the form of configuration or setup.	○	○	○	○	○
9. Synchronization toolkits and data retrieval mechanisms are created to respond to the demands posed by integration.	○	○	○	○	○

SaaS Modification

Modification refers to techniques and solutions that alter the application design and other functional requirements of the application by means of alterations implemented to the source code.

Please indicate the extent to which you feel the following statements represent the **modification approach** in the SaaS Multi-Tenant context.

Questions	Strongly Disagree	Disagree	Neither Nor	Agree	Strongly agree
1. Source code modifications are made to SaaS application to generate a new functionality without changing a shared code base.	○	○	○	○	○
2. The code modification must take resources allocation for customized code into account, ensuring operational cost-efficiency in terms of maintenance costs and resource sharing among tenants.	○	○	○	○	○
3. SaaS vendors must manage all elements of customization codes on an individual tenant basis without developing many software versions for each tenant.	○	○	○	○	○
4. SaaS vendors alter application codes when identical customizations are defined and justified by a considerable number of tenants.	○	○	○	○	○
5. Source code modifications are made by adding/deleting methods or attributes, or by changing the object's current implementation methods.	○	○	○	○	○

Part 3: SaaS Quality

Based on the definition provided for each quality attribute, please indicate the extent to which you feel each quality attribute plays an important role in customization.

	Questions	Strongly Disagree	Disagree	Neither Nor	Agree	Strongly agree
1.	**Multi-tenancy:** SaaS services can support instances of simultaneous access by multiple users for multiple tenants.	○	○	○	○	○
2.	**Scalability:** SaaS providers can manage growth or decline in the level of services.	○	○	○	○	○
3.	**Availability:** SaaS services can function within a specific time to satisfy users' needs.	○	○	○	○	○
4.	**Reliability:** SaaS application maintains operating and functioning under given conditions without failure within a given time period.	○	○	○	○	○
5.	**Maintainability:** Modifications to the application are made by SaaS provider to retain it in the condition of good repair.	○	○	○	○	○
6.	**Security:** the effectiveness of SaaS provider's controls on service data, access to the services, and the physical facilities from which service are provided.	○	○	○	○	○
7.	**Usability:** the ease with which SaaS application can be used to achieve tenant-specific-goal.	○	○	○	○	○
8.	**Interoperability:** SaaS service can easily interact with other services from the same SaaS provider or other providers.	○	○	○	○	○
9.	**Efficiency**: SaaS services effectively utilize resources to perform their functions.	○	○	○	○	○
10.	**Functionality:** SaaS application provides an extensive set of features.	○	○	○	○	○
11.	**Accessibility:** SaaS services are operable by users with different disabilities.	○	○	○	○	○
12.	**Commonality:** SaaS services possess common features and are amenable to reuse by multiple users.	○	○	○	○	○
13.	**Response time:** SaaS application adheres to a defined time limit between service request and service response.	○	○	○	○	○

Thank you for your time
